# 1,3-Diarylpyrazolyl-acylsulfonamides Target HadAB/BC Complex in *Mycobacterium tuberculosis*

**DOI:** 10.1021/acsinfecdis.2c00392

**Published:** 2022-11-03

**Authors:** Vinayak Singh, Anna E. Grzegorzewicz, Stephen Fienberg, Rudolf Müller, Lutete Peguy Khonde, Olalla Sanz, Salvatore Alfonso, Beatriz Urones, Gerard Drewes, Marcus Bantscheff, Sonja Ghidelli-Disse, Thomas R. Ioerger, Bhanupriya Angala, Jiuyu Liu, Richard E. Lee, James C. Sacchettini, Inna V. Krieger, Mary Jackson, Kelly Chibale, Sandeep R. Ghorpade

**Affiliations:** † Drug Discovery and Development Centre (H3D), University of Cape Town, Rondebosch7701, South Africa; ‡ South African Medical Research Council Drug Discovery and Development Research Unit, Department of Chemistry and Institute of Infectious Disease and Molecular Medicine, University of Cape Town, Rondebosch7701, South Africa; § Mycobacteria Research Laboratories, Department of Microbiology, Immunology and Pathology, Colorado State University, Fort Collins, Colorado80523-1682, United States;; ∥ Global Health Pharma Research Unit, GlaxoSmithKline, Severo Ochoa 2, Tres Cantos, Madrid28760, Spain; ⊥ Cellzome GmbH · A GSK Company, Meyerhofstrasse 1, Heidelberg69117, Germany; # Department of Computer Science and Engineering, Texas A&M University, College Station, Texas77843, United States; g Department of Chemical Biology & Therapeutics, St. Jude Children’s Research Hospital, 262 Danny Thomas Place, Memphis, Tennessee38105, United States;; h Texas A&M University, Department of Biochemistry and Biophysics, ILSB 2138, 301 Old Main Dr, College Station, Texas77843-3474, United States

**Keywords:** *Mycobacterium tuberculosis*, tuberculosis drug discovery, 3-hydroxyl-ACP dehydratase, 1,3-diarylpyrazolyl-acylsulfonamides, FASII pathway

## Abstract

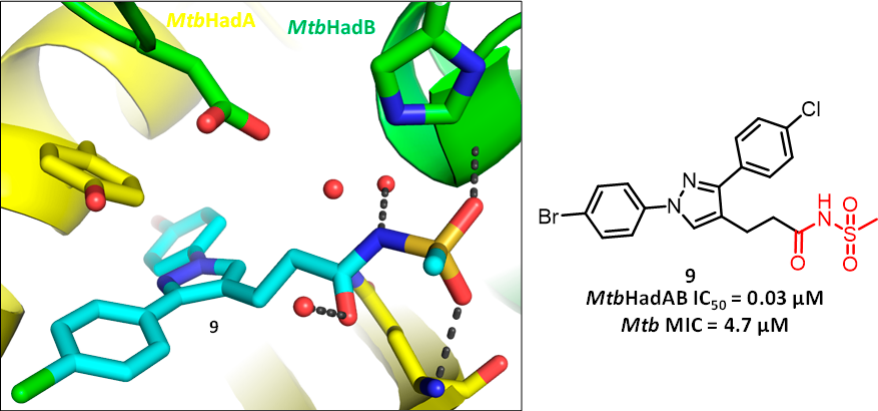

Alternative mode-of-inhibition of clinically validated targets is an effective strategy for circumventing existing clinical drug resistance. Herein, we report 1,3-diarylpyrazolyl-acylsulfonamides as potent inhibitors of HadAB/BC, a 3-hydroxyl-ACP dehydratase complex required to iteratively elongate the meromycolate chain of mycolic acids in *Mycobacterium tuberculosis* (*Mtb*). Mutations in compound **1**-resistant *Mtb* mutants mapped to HadC (Rv0637; K157R), while chemoproteomics confirmed the compound’s binding to HadA (Rv0635), HadB (Rv0636), and HadC. The compounds effectively inhibited the HadAB and HadBC enzyme activities and affected mycolic acid biosynthesis in *Mtb*, in a concentration-dependent manner. Unlike known 3-hydroxyl-ACP dehydratase complex inhibitors of clinical significance, isoxyl and thioacetazone, 1,3-diarylpyrazolyl-acylsulfonamides did not require activation by EthA and thus are not liable to EthA-mediated resistance. Further, the crystal structure of a key compound in a complex with *Mtb* HadAB revealed unique binding interactions within the active site of HadAB, providing a useful tool for further structure-based optimization of the series.

In the years leading up to the Covid-19 pandemic, tuberculosis (TB), which is caused by a single infectious pathogen, *Mycobacterium tuberculosis* (*Mtb*), was the most deadly infectious disease in the world.^1^ During this time, the global number of TB cases continued to rise and was mainly fueled by poverty, the HIV/AIDS coinfection, and the emergence of multidrug-resistant and extensively drug-resistant strains of *Mtb*. While significant progress has been made in treating drug-resistant TB by introducing bedaquiline (BDQ) and working up new regimens, it is insufficient to meet current and projected therapeutic needs, and requires new lead chemical entities entering drug discovery pipelines.^2^ Therefore, there is a clear need for drugs with novel modes of action (MoA) or alternative mode of inhibition (MoI) of a clinically validated target showing no cross-resistance to currently used drugs to aid in the fight against TB.

The cell envelope of *Mtb* is one of the most attractive targets in TB drug discovery. Mycolic acids, which are unique to mycobacteria, play a crucial role in the integrity and maintenance of the cell envelope.^3,4^ Indeed, cell wall biosynthesis is a clinically validated target for TB drug discovery, as exemplified by the successful clinical use of a number of past and present anti-TB drugs such as isoniazid (INH), ethionamide (ETH), thioacetazone (TAC), and isoxyl (ISO).^5,6^ It is noteworthy that while the biosynthesis and export of mycolic acids involve over 30 proteins,^7,8^ inhibitors that have made it to the clinic thus far only target two biosynthetic steps, both of which are located within the type II fatty acid synthase (FAS-II) pathway.^9^ FAS-II, required to iteratively elongate the main meromycolate chain of mycolic acids, is composed of four dissociable sets of enzymes: the reductase MabA, the dehydratases HadAB and HadBC, the enoyl-ACP reductase InhA, and the condensases KasA and KasB (Figure 1). INH and ETH inhibit the enoyl-ACP reductase InhA, whereas ISO and TAC target the essential dehydratase HadAB.^10^ A recent study reported that the two inhibitors targeting different steps of FAS-II (KasA and InhA) used in combination not only display synergistic lethality *in vitro* and *in vivo* but also have the ability to kill persistent, nonreplicating *Mtb*, which provides a new incentive to develop inhibitors of the other steps of the FAS-II elongation system.^11^

**Figure 1:**
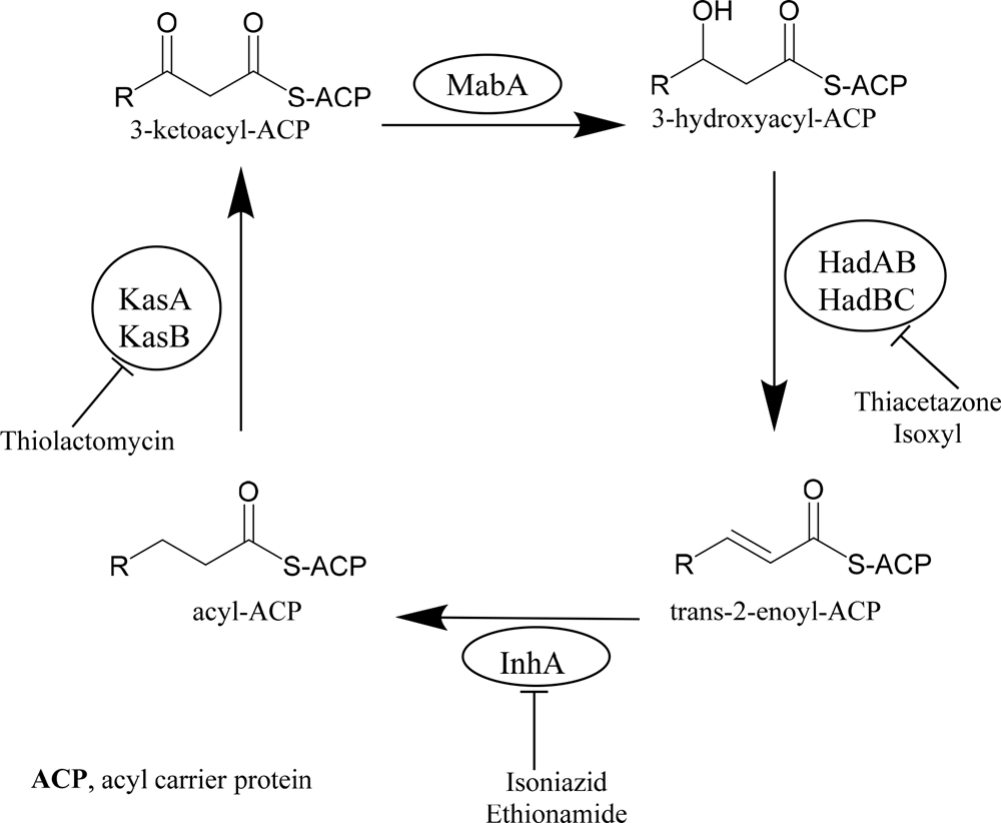
Fatty acid synthase (FAS-II) pathway in *Mycobacterium tuberculosis*.

Recently, we reported a 1,3-diarylpyrazolyl-acylsulfonamide chemical series with potent anti-*Mtb* activity identified from the phenotypic screening of a large compound library.^12^ Preliminary MoA studies demonstrated that compounds from the series targeted cell wall biosynthesis but *Mtb* mutants of common cell wall targets such as MmpL3, DprE1, InhA, and EthA did not show resistance to this series. Here, by using various chemical biology approaches, we identified that 1,3-diarylpyrazolyl-acylsulfonamides target the 3-hydroxyl-ACP dehydratase complex, HadAB/BC. We further confirm that the compounds interact uniquely with HadAB/BC complex and avoid the EthA-related mechanism of activation. Together, these data validate the MoA of 1,3-diarylpyrazolyl-acylsulfonamides in *Mtb*.

## Results and Discussion

### Initial Microbiological Characterization of the Compounds

Table 1 summarizes *Mtb* whole-cell activities of compounds **1** and **2** that are used as tool compounds for MoA studies. Compound **1** with MIC of 0.2 μM was approximately 4-fold more potent than **2**, but both compounds had a similar order of minimum inhibitory concentrations (MICs) against *Mtb* H37Rv in glucose as well as in cholesterol containing Middlebrook 7H9 media (7H9/glucose/BSA/Tx and 7H9/DPCC/cholesterol/BSA/Tx) and showed 4–6-fold better activity under serum-free conditions (7H9/casitone/Tx or 7H9/DPCC/casitone/Tx). The compounds were bactericidal against replicating *Mtb* and retained potency against both drug susceptible and resistant clinical isolates of *Mtb.*(12) Compounds were screened against a collection of Gram-negative and one Gram-positive bacteria (ESKAPE) to determine the spectrum of activity/selectivity and were found to be inactive against Gram-negative pathogens (*Enterobacter cloacae*, *Escherichia coli*, *Klebsiella pneumoniae*, *Acinetobacter baumannii*, and *Pseudomonas aeruginosa*) when tested up to a concentration of 128 μM (Table S1). Compound **2** displayed a weak activity (MIC, 128 μM) against Gram-positive *Staphylococcus aureus*. This confirmed the selectivity of 1,3-diarylpyrazolyl-acylsulfonamides for *Mtb* and encouraged further progression of these compounds.

**Table 1:**
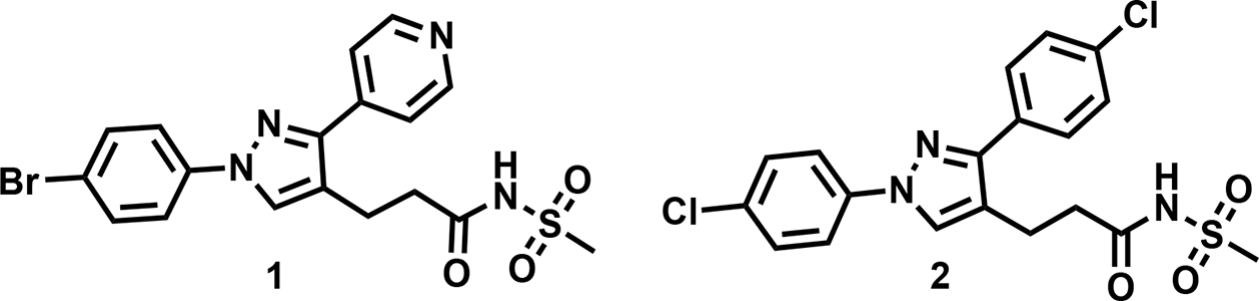
*Mtb* Whole-Cell Activities of **1** and **2**

properties^a^	**1** (μM)	**2** (μM)
*Mtb* MIC (Middlebrook 7H9/casitone/Tx)	0.2	0.6
*Mtb* MIC (Middlebrook 7H9/glucose/BSA/Tx)	1.2	4.7
*Mtb* MIC (Middlebrook 7H9/DPPC/cholesterol/BSA/Tx)	0.8	4.7
*Mtb* MIC (Middlebrook 7H9/DPCC/casitone/Tx)	0.3	0.8
*Mtb* MBC (Middlebrook 7H9/glucose/ADC/Tx)	7.6	10
HepG2 IC_50_ (glucose)	>50	>50
HepG2 IC_50_ (galactose)	>50	>50
solubility	190	100

a Tx, tyloxapol; BSA, bovine serum albumin; DPPC, dipalmitoylphosphatidylcholine; ADC, albumin dextrose catalase; MIC, the lowest concentration of the given compound that inhibits >90% *Mtb* growth; MBC, the concentration of the given compound that showed ≥2 Log kill of the *Mtb* population.

### Mechanism of Action Studies

#### Isolation and Characterization of Compound **1**-Resistant Mutants of *Mtb*

As previously mentioned,^12^ preliminary MoA results indicated cell wall biosynthesis as a target. It was based on the sustained signals in the P*iniB*-LUX bioluminescence reporter assay,^13^ detecting modulation in the expression of *iniBAC* operon, which is an indicator of cell wall damage, and transcriptional profiling studies that showed upregulation of the genes involved in the cell wall biosynthesis. The compounds were active against *Mtb* mutants of known cell wall targets, suggesting the potential involvement of a unique MoA. As a first step toward target identification, we decided to generate spontaneous-resistant mutants (SRMs) to compound **1** as a representative compound of this series. SRMs against compound **1** were selected by plating *Mtb* cells on Middlebrook 7H10 agar supplemented with glycerol and oleic acid/albumin/dextrose/catalase (OADC), containing **1** at 3×MIC, 5×MIC, 10×MIC, or 20×MIC concentrations (1×MIC = 1.9 μM, in Middlebrook 7H9/glycerol/albumin-dextrose-catalase (ADC)/tween-80). Mutants were only obtained from the 3×MIC plate at a frequency of approximately 1.9 × 10^–8^. There was no growth observed on the plates containing a higher compound concentration. Ten individual colonies were picked, grown (in absence of compound) in Middlebrook 7H9/glycerol/ADC/tween-80, and retested for susceptibility to **1**. Only seven (out of 10) of the selected colonies displayed reproducible phenotypic resistance (MIC, 50–100 μM) to **1**, suggesting that the frequency of resistance observed from the original plating overestimated the actual frequency of heritable resistance (Table S2). Three random SRMs were selected for whole-genome sequence analyses compared to the parental wild-type *Mtb* H37Rv strain. The analyses revealed two nonsynonymous single nucleotide polymorphisms (nsSNPs) in all three mutants, confirmed by Sanger sequencing (Table S2). All mutant strains carried a nsSNP (K157R) in a nonessential gene, *hadC* (*Rv0637c*), a component of the HadBC dehydratase involved in FAS-II.^14^ The dehydratase step of FAS-II involves two separate enzymes, HadAB and HadBC, sharing the same catalytic subunit, HadB. Interestingly, HadAB is essential for growth, whereas HadBC is not, consistent with HadAB acting on shorter meromycolate precursors during the early stages of their elongation by FAS-II and HadBC acting at later stages on longer fatty acyl substrates.^14^ The K157R point mutation in HadC was recently shown to permit HadBC to functionally compensate for HadAB.^15^ Collectively, this finding of resistance nsSNP and the bactericidal activity of the compounds suggests that they inhibit HadAB inside the cells. All the mutants also carried a nsSNP in nonessential protein *ppsB*, which encodes a type I polyketide synthase involved in phenolphthiocerol and phthiocerol dimycoserosate (PDIM) biosynthesis. The PDIM biosynthetic capacity is commonly lost during propagation of *Mtb in vitro*; this leads to the conclusion that the *ppsB* mutation was unlikely to significantly contribute to the resistance. To support this, we tested **1** against a PDIM-deficient H37Rv strain (H37RvJO), and the observed activity was similar to the PDIM producing strain (H37RvMA).

Prodrugs TAC and ISO, known to target HadAB, require *S*-oxidation of the thiocarbonyl moiety by the flavin-containing monooxygenase EthA intracellularly in order to inhibit the enzyme. Compounds **1** and **2** showed no change in the MICs against the EthA mutant (C253R) of *Mtb* that is 4–8 folds resistant to TAC and ISO, ruling out the activation of the compounds by EthA.^12^

#### Chemoproteomics Identified Both HadAB and HadBC As Possible Targets

**Figure 2:**
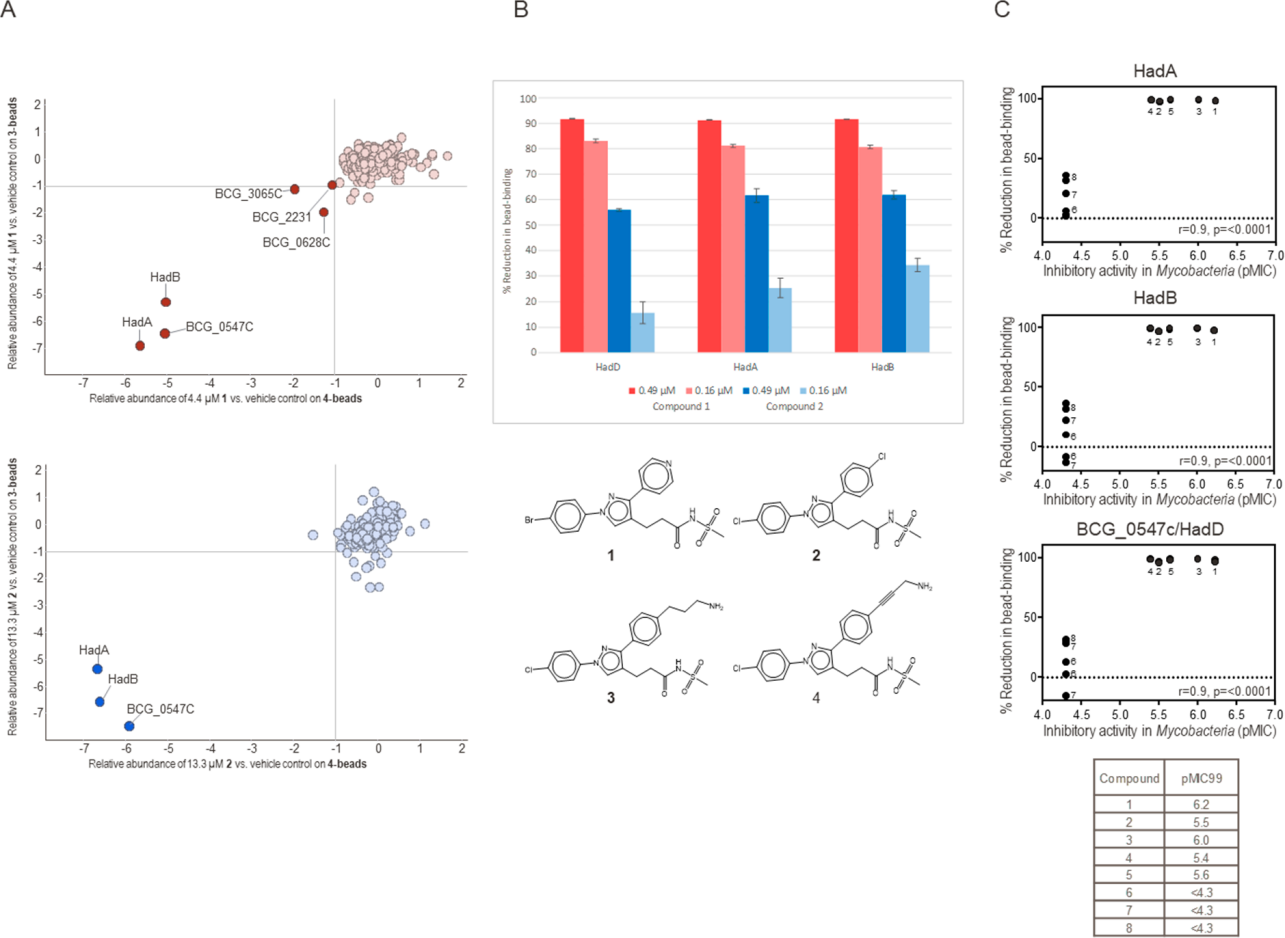
Chemoproteomic profiling of **1** and **2** identified HadA, HadB, and BCG_0547c (HadD) as targets. (A) Compounds **2** (blue) or **1** (red) were analyzed in a *Mycobacterium bovis BCG* protein extract with compound **3** and **4** attached to a bead matrix. Only three proteins showed competition by both compounds, HadA, HadB, and BCG_0547c (HadD). (B) Compound **1** (red) showed stronger inhibition of all three proteins from bead-binding in comparison to compound **2** (blue), indicating higher potency (*n* = 3). (C) Correlation of target binding and antimycobacterial activity of compounds from the 1,3-diarylpyrazolyl-acylsulfonamide series. Active compounds (**1**-**5**) showed inhibition of proteins HadA, HadB, and BCG_0547c (HadD) from **4**-bead-binding, whereas inactive compounds (**6**–**8**) did not (results on **3**-beads are comparable, Figure S1). Numbers indicate compounds. *r*, Pearson correlation coefficient; *p*, *p*-value (calculated probability); pMIC99 defined as −log10(*Mtb* MIC_99_ in M).

Next, in our MoA deconvolution efforts, compounds **1** and **2** were analyzed by affinity-enrichment chemoproteomics to identify the protein targets of the series. Two amine-functionalized analogues, **3** and **4** (Figure 2A), were immobilized on sepharose beads. A quantitative competition-based approach was applied to distinguish between proteins binding to the immobilized compound and the background caused by proteins binding directly to the sepharose matrix. The compounds, **1** or **2**, were spiked into aliquots of protein extract generated from *Mycobacterium bovis* BCG over a range of concentrations and competed with the immobilized analogue for binding to the target proteins. Matrix-bound proteins were eluted, trypsinized, and subsequently encoded with isobaric mass tags (TMT10), enabling relative quantification by LC-MS/MS. Target proteins of the test compounds were prevented from bead-binding in a dose-dependent manner, thus allowing the determination of half-maximal binding concentrations (IC_50_). Apparent dissociation constants (*K*_d_^app^) were derived from the IC_50_ values by taking into account the amount of target sequestered by the affinity-matrix using the Cheng-Prusoff relationship (IC_50_/*K*_d_^app^ correction factor) and sequential binding experiments.^16^ Duplicate solvent controls, sequential binding experiments, and a 6-point dose–response (40–0.16 μM, 1:3 dilutions) were analyzed in a single 10-plexed mass spectrometric experiment.^17^

Test compounds **1** and **2** were analyzed on both bead matrices, **3**- and **4**-beads, in three independent experiments. The addition of both compounds resulted in a reduction of amount in the bead-bound fraction of three proteins: HadA, HadB, and BCG_0547c; these proteins were considered as target candidates for the series (Figure 2A). BCG_0547c is an orthologue to MSMEG_0948 and Rv0504c. HadD_*Mtb*_ (Rv0504c) shares a sequence identity of 63% with HadD_*Msm*_ (MSMEG_0948) (68% using BlastP alignment).^18^ To confirm BCG_0547c to be HadD in *M. bovis* BCG, we generated a sequence alignment using BlastP. The HadD_*Mtb*_ (Rv0504c) displayed a sequence identity of 100% with BCG_0547c (not shown); hence BCG_0547c will be referred to as HadD in the following text. HadD_*Mtb*_ appears to catalyze the 3-hydroxyacyl dehydration step of late FAS-II elongation cycles during keto-MA biosynthesis.^19^ MSMEG_0948 (HadD) protein from *M*. *smegmatis* interacts with the FAS-II heterodimeric dehydratase HadA-HadB (HadAB).^18^ Chemoproteomic profiling results were consistent with HadA, HadB, and HadD interacting with immobilized compounds as a protein complex, as all three proteins showed almost identical values for the competition from the bead matrices for compound **1** (91–92% at 0.49 μM), and **2** (56–62% at 0.49 μM), respectively (Figure 2B). The calculated IC_50_ values were below the assay window of 0.49 μM for compound **2** and below 0.16 μM for compound **1** for all three proteins, respectively. Compound **1** showed additional competition of three proteins, albeit to a lower degree compared to HadA/B/D. An apparent *K*_d_ value of 1.0 μM was generated for **1** and BCG_2231 (dihydrolipoamide acetyltransferase component of pyruvate dehydrogenase complex, DlaT, Rv2215, it was also competed by **2**, with a *K*_d_^app^ value of 6.6 μM). The *K*_d_^app^ value for **1** and BCG_0628c (probable conserved lipoprotein lpqN, Rv0583c) was 1.8 μM, and for BCG_3065c (probable conserved ATP-binding protein ABC transporter, Rv3041c), the *K*_d_^app^ value was 0.4 μM (for additional details, see Table S3 and Figure S1).

To analyze whether or not HadA, HadB, and HadD were primary targets of the compounds from the series, we used eight compounds—five active (compounds **1**–**5**) and three inactive (compounds **6**–**8**) against *Mtb*—in chemoproteomics profiling experiments at 10 μM in duplicate to correlate target binding with antimycobactericidal activity (Figure 2C; Table S3). The correlation plots show a good correlation with a Pearson correlation coefficient of 0.9 and *p*-values of less than 0.0001 (Figure 2C). Generally, at 10 μM, all five active compounds showed nearly complete prevention of all three proteins from bead binding, while this was not the case with inactive compounds. This was seen on both bead matrices, **3** and **4**. Interestingly, HadC was identified and competed by active compounds from bead binding but not by inactive compounds (Figure S1). But the HadC inhibition from bead binding at a 10 μM compound concentration was between 52 and 64%, while the inhibition for the complex HadA/B/D in the same experiments was in the range of 94–100% (Figure S1), which indicates higher IC_50_ values for HadC compared to the HadA/B/D-complex (no IC_50_ values were generated for HadC in these experiments).

### Diarylpyrazolyl-acylsulfonamides Inhibit Both HadAB and HadBC

To assess whether or not HadAB and HadBC are the target(s) of 1,3-diarylpyrazolyl-acylsulfonamides, we determined the inhibitory activity of compounds with a diverse range of *Mtb* MICs against both dehydratases *in vitro* using a previously described enzymatic assay.^14^ This assay employs *Mtb* HadAB or HadBC enzymatic complexes expressed and purified from *E. coli*, and *trans*-2-hexadecenoyl-CoA (*trans*-2-C_16:1_-CoA) as the substrate. Enzymatic assays revealed significant inhibition of HadAB enzymatic complex, with the most effective compounds’ IC_50_ values ranging from 0.018 to 4.2 μM. HadAB enzyme inhibition potency correlated well with *Mtb* MICs (Figure S2 scatter plot). While most acyl sulfonamides (**2**, **3**) did not inhibit HadBC enzyme activity at 50 μM, acyl sulfonylureas (**11** and **14**) and oxadiazolone **12** showed moderate inhibition of HadBC at 50 μM (Table 2). These results suggest that inhibition of HadAB rather than HadD likely accounts for the bactericidal activity of the compounds since HadD is nonessential.^19^ This is in agreement with a K157R mutation in HadC providing resistance to the compounds as it permits HadBC to functionally compensate for HadAB while being a lot less susceptible to the inhibition by these compounds.^15^

**Table 2:**
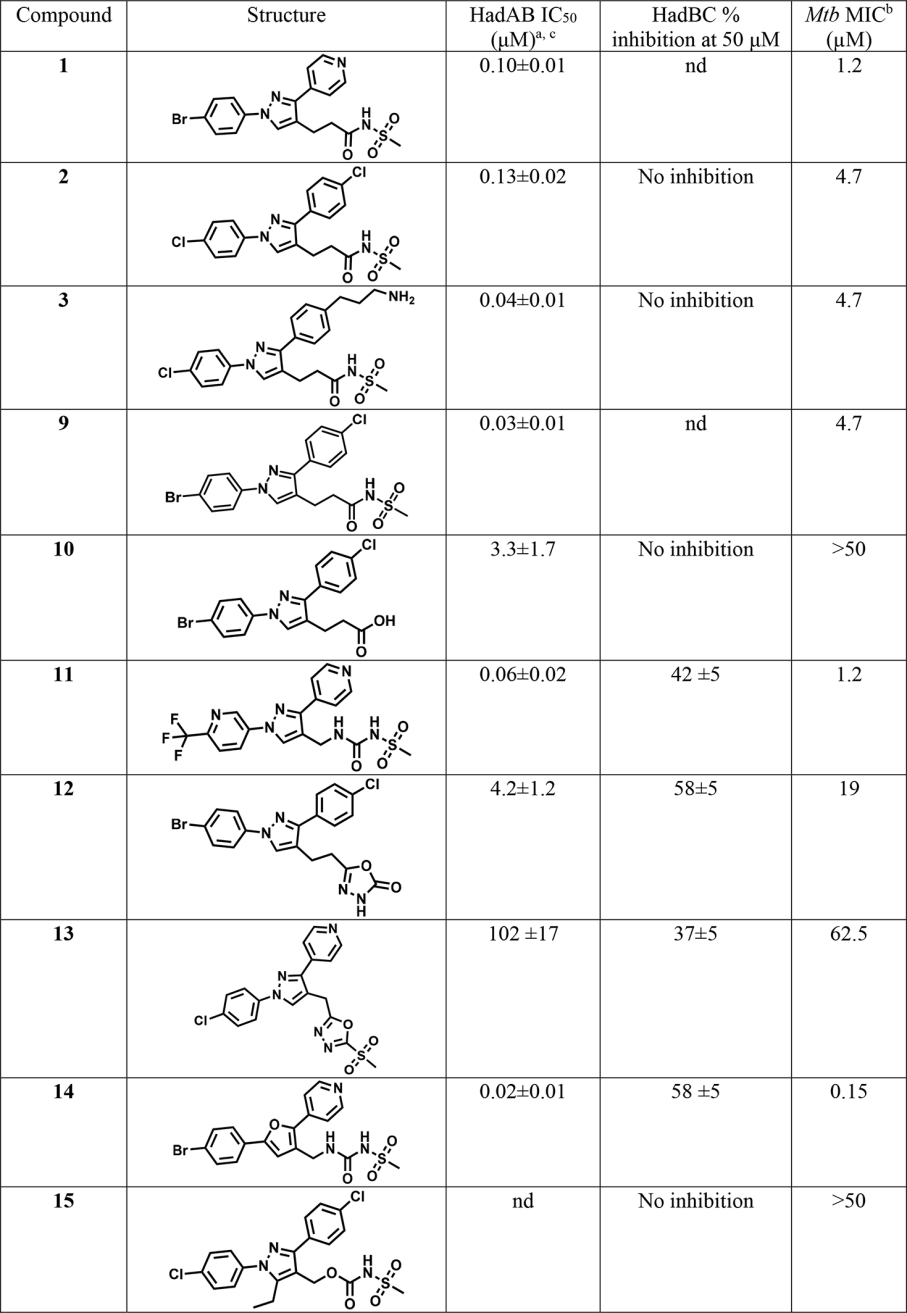
Inhibition of HadAB and HadBC Enzyme Complexes by 1,3-Diarylpyrazolyl-acylsulfonamides

a Inhibition values of 1,3-diarylpyrazolyl-acylsulfonamides (50 μM) for HadAB (22 nM) and HadBC (130 nM) as measured with *trans*-2-C_16:1_-CoA substrate (60 μM in the HadAB assay; 25 μM in the HadBC assay) using the spectrophotometric assay as described previously.^14^

b MIC in Middlebrook 7H9/glucose/BSA/Tx). IC_50_ values of the selected compounds for HadAB enzymatic complex were determined using *GraFit* (Version 5.0.13). Hill slopes were not constrained, ranging between 1–2.5. Compounds with solubility issues displayed greater variability in testing and resulted in higher Hill slopes upon IC_50_ curve fitting.

c IC_50_ values are close to the enzyme concentration used in the assay (22 nM) and could be more potent than the calculated value. Data are means ± standard deviations of duplicate assays and represent two separate trials; nd, not determined.

### 1,3-Diarylpyrazolyl-acylsulfonamides Inhibit Mycolic Acids Biosynthesis

**Figure 3:**
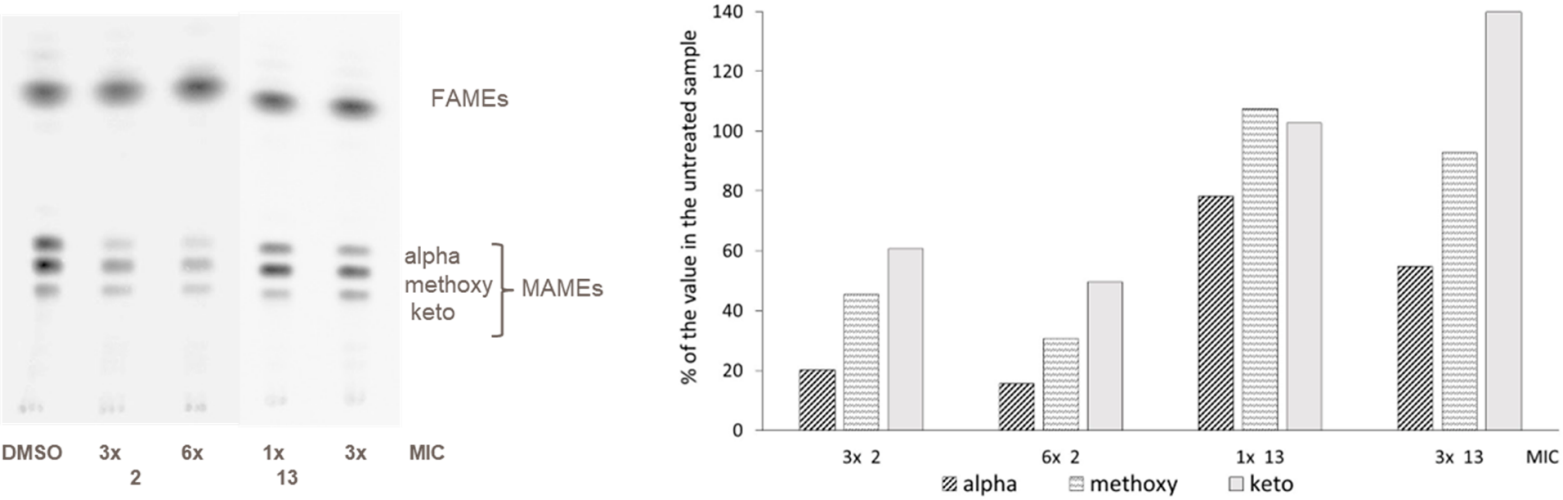
Effect of 1,3-diarylpyrazolyl-acylsulfonamides on mycolic acid biosynthesis in *Mycobacterium tuberculosis*. *Mtb* H37Rv mc^2^6206 grown in Middlebrook 7H9-OADC-tyloxapol supplemented with casamino acids, pantothenate, and l-leucine at 37 °C was treated with no drug or **2** or **13** (1 to 6× MIC) and metabolically labeled with [1,2-^14^C]-acetate for 24 h. The same volume of [^14^C]-acetate-labeled fatty acid and mycolic acid methyl esters (FAMEs and MAMEs) from treated and untreated cells were analyzed by TLC in the solvent system [*n*-hexanes/ethyl acetate 95:5; by vol.; three developments] and revealed by PhosphorImaging. Compound **2**, a potent HadAB inhibitor, inhibited biosynthesis of mycolic acids up to 86% at 3× MIC in 24 h, whereas only 0–49% inhibition was observed with a weaker HadAB inhibitor **13** at 3× MIC.

To investigate the effect of 1,3-diarylpyrazolyl-acylsulfonamides on the end products of this biosynthetic pathway, the mycolic acids, we labeled compound-treated and untreated *Mtb* H37Rv mc^2^6206 (an avirulent Δ*panCD*Δ*leuCD* mutant of *Mtb* H37Rv) with [^14^C]-acetate and analyzed their mycolic acid methyl ester (MAME) profiles by TLC (Figure 3; see Table S4 for MICs against *Mtb* H37Rv mc^2^6206). Exposure of *Mtb* to the 1,3-diarylpyrazolyl-acylsulfonamides led to a concentration-dependent decrease in *de novo* mycolic acid synthesis. The most effective compound (compound **2** at 6×MIC) inhibited biosynthesis of mycolic acids up to 86% in 24 h. Though all three classes of mycolic acids were affected by treatment, the most dramatic effect was observed for alpha mycolates. Collectively, the enzyme assays and the mycolic acid profiles consistent with the inhibition of enzymatic activities of HadAB/BC validate the FAS-II pathway as the target of 1,3-diarylpyrazolyl-acylsulfonamides.

### 1,3-Diarylpyrazolyl-acylsulfonamides Inhibit *Mtb* Growth by Targeting HadAB/HadBC

To further confirm the on-target activity of 1,3-diarylpyrazolyl-acylsulfonamides in whole *Mtb* bacilli, we investigated whether the overexpression of the *hadABC* operon, previously shown to confer high-level resistance to ISO and TAC, would decrease the sensitivity to 1,3-diarylpyrazolyl-acylsulfonamides. Compounds were tested for MICs against *Mtb* strain overexpressing *hadABC* relative to the parent strain, *Mtb* H37Rv mc^2^6206. The increase in MICs (2 to 64-fold) for all the compounds except **13** confirmed the reduced susceptibility of the *hadABC* overexpressor to 1,3-diarylpyrazolyl-acylsulfonamides. This further supports the conclusion that 1,3-diarylpyrazolyl-acylsulfonamides target the 3-hydroxyl-ACP dehydratase complex (Table S4).

### HadAB 1,3-Diarylpyrazolyl-acylsulfonamide Cocrystal Structure

**Figure 4:**
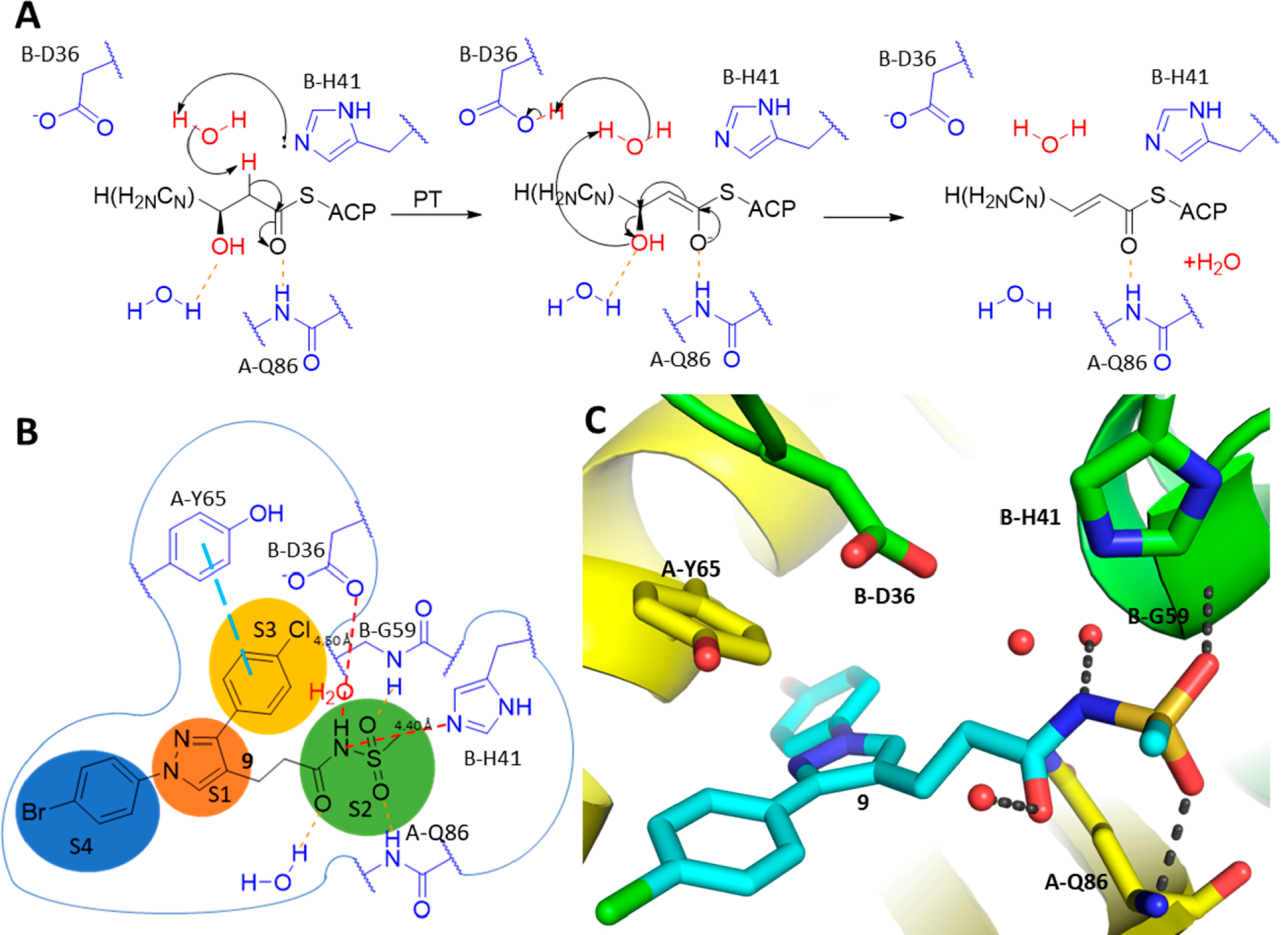
Acyl sulfonamide inhibitor **9** binds to the active site of *Mtb* HadAB, mimicking the high energy oxyanion intermediate of the fatty acid-ACP dehydratase reaction. (A) Generalized fatty acid-ACP dehydratase mechanism facilitated in the binding site of the HadAB complex. Substrate binding is facilitated by hydrogen bonds to the backbone amide of A-Q86 and a fixed water. (B) 2D schematic of the HadAB binding site. Here four subsites are defined as S1, S2, S3, and S4, respectively, each accommodating a specific group from the bound ligand. The acyl sulfonamide moiety binding to the S2 subsite mimics the H-bonding pattern of the high energy oxyanion intermediate of the fatty acid-ACP dehydratase reaction, with the enolizable sulfonamide N equidistant (4.4–4.5 Å; red dashed lines) from the centers of the basic N of B-H41 and an O of B-D36, ideally located for a water-mediated deprotonation and proton transfer. The S3 subsite leads to an opening of the HadAB heterodimeric complex toward solvent. This channel features a prominent aromatic residue B-Y65, forming a π-stacking interaction with the chlorophenyl group. S1 is a nonspecific subsite that fits several heterocyclic cores including pyrazole. S4 is a hydrophobic cavity bound by nonaromatic lipophilic residues like B-L91 and B-I60. It binds large hydrophobic groups like the bromophenyl moiety. (C) 3D illustration of the binding mode of **9** interacting with the HadAB complex with the HadA subunit depicted in yellow and the HadB subunit depicted in green. Ligand **9** is depicted in cyan and the H-bonds are depicted in gray. This view focuses on the H-bond interactions between the acyl sulfonamide moiety of **9** and the backbone amides of A-Q86, B-G59, and a fixed water. A catalytic water is trapped between the catalytic B-D36 and B-H41 residues, bridging them via H-bonds. A-Y65 and the chlorophenyl moiety are also shown to be lined up with π-stacking interactions, while the bromophenyl moiety is shown to extend into a lipophilic pocket toward the back of this view (PDB ID: 7SVT).

To elucidate the binding mode of acyl sulfonamides to *Mtb* HadAB, compound **9** was cocrystallized with the *Mtb* HadAB complex (PDB ID: 7SVT). The complex crystallized in *P2*_1_2_1_2_1_ space group with 4 HadAB complexes in the asymmetric unit. Each one of the four active sites showed a strong electron density for the inhibitor (Figure S3). The cocrystal structure of **9** is the first published HadAB inhibitor that binds to the active site by engaging the catalytic dyad of D36 and H41 of the HadB subunit, mimicking the high energy oxyanion intermediate of the fatty acid-ACP dehydratase reaction (amino acid residues on HadA prefixed as A- and those on HadB as B-) (Figure 4A).

The fatty acid-ACP binds to the HadAB complex forming H-bonds with the amide of A-Q86 and a water molecule.^20^ B-H41 then deprotonates the α carbon via a catalytic water and goes on to create a high energy oxyanion intermediate that is stabilized by a H-bond with A-Q86 while the proton is transferred to B-D36 and back to the hydroxyl leaving group via the same catalytic water as the oxyanion enolate returns to its carbonyl form, creating an α–β-unsaturated bond with the expulsion of a water molecule. The acyl sulfonamide moiety of the inhibitor mimics this high energy oxyanion intermediate of this enzyme catalyzed dehydratase reaction; hence it is a key driver of *Mtb* HadAB inhibition by the compounds in the series.

The *Mtb* HadAB binding site may be divided into four subsites, S1, S2, S3, and S4 (Figure 4B), to facilitate the discussion. The S1 subsite is hydrophobic and close to three aromatic residues from both the HadA and HadB subunits, favoring small rings with π-character, allowing it to accommodate the heteroaromatic pyrazole core. The S2 subsite is the site of catalysis for the dehydratase reaction on the interface of the two subunits. It binds the acyl sulfone as a mimic of the high energy oxyanion intermediate of the dehydratase reaction. Here, the sulfone forms hydrogen bonds with backbone amides from both subunits (A-Q86 and B-G59), and the carbonyl oxygen of the inhibitor is stabilized via a hydrogen bond to a water in the catalytic site. The S3 subsite is characterized by lipophilic aromatic residues like A-Y65, well suited to bind the aromatic chlorophenyl moiety. The S4 subsite, a large hydrophobic cavity with no aromatic residues; accommodates the bromophenyl moiety. This binding mode of a trisubstituted pyrazole with the acyl sulfonamide mimicking a catalytic intermediate in the S2 subsite (Figure 4C) provides valuable insights allowing for the rationalization of SAR observed for this series, laying a foundation for future structure-based design.

The crystal structure showing an inhibitor engaging the catalytic site residues of *Mtb* HadAB is an important achievement, as clinically significant inhibitors of *Mtb* HadAB ISO and TAC cannot be directly complexed with the enzyme as they require intracellular activation, and relatively weak *Mtb* HadAB inhibitors with cocrystal structures available, butein and fisetin, with respective *K*_i_ values of 13.5 and 10.9 μM,^21^ only occupy the S1 and S4 subsites, and not the S2 catalytic subsite (Figure S4, PDB IDs 4RLW and 4RLT, respectively).

### Docking of 1,3-Diarylpyrazolyl-acylsulfonamides into *Mtb* HadAB Supports the SAR

A *Mtb* HadAB active site docking grid was prepared based on the binding site of **9** retaining the bound water molecule in the active site. Compound **9** was then docked into this docking grid using Glide with a result that closely reproduced the crystal structure pose with an all atom RMSD of 5.92 Å. With an experimentally validated docking pose for the 1,3-diarylpyrazolyl-acylsulfonamide in the *Mtb* HadAB complex, the observed structure–activity relationship of this series could be rationalized through the docking of other compounds from the series and serve as a foundation for the further design.

**Figure 5:**
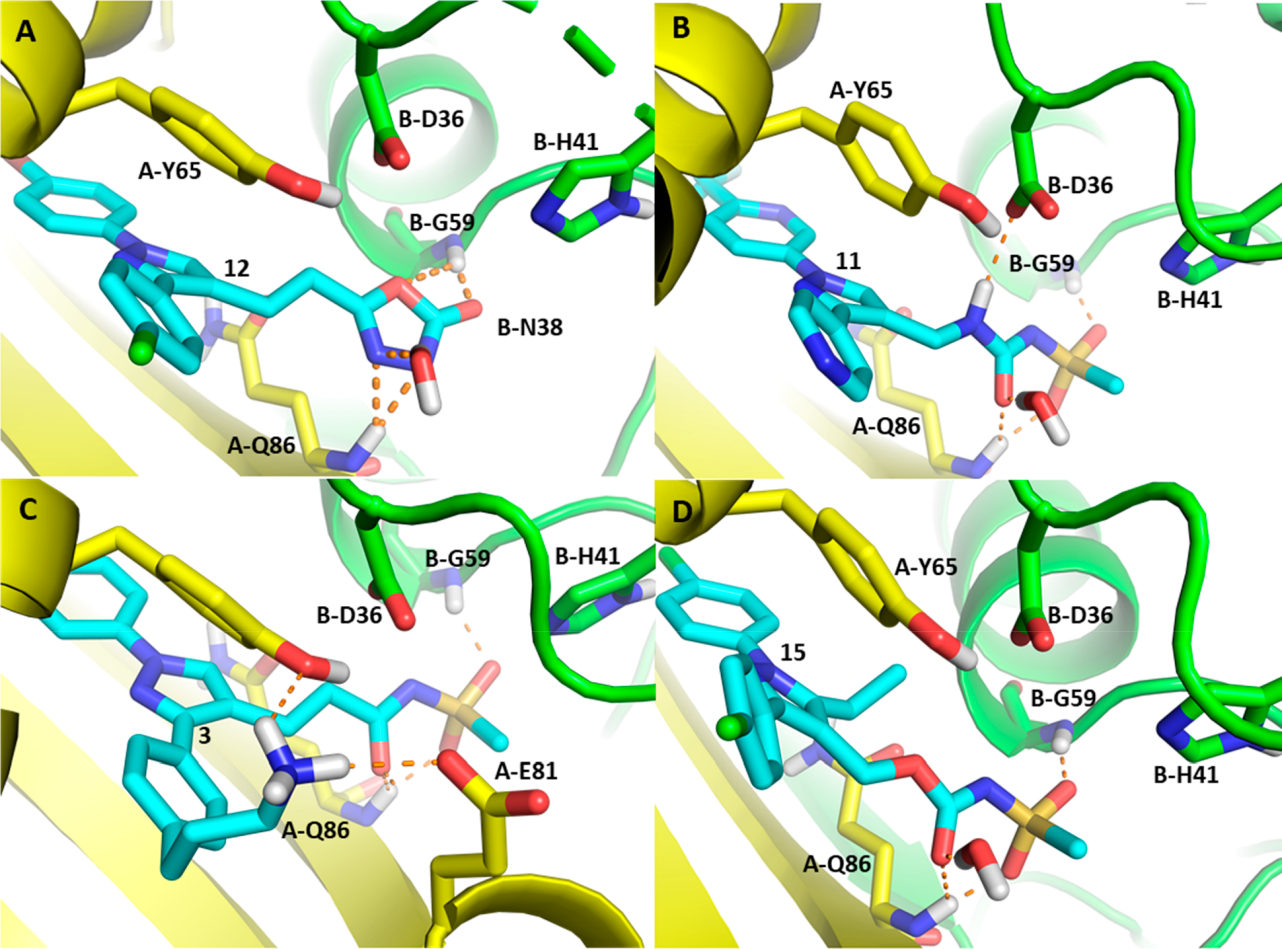
Docking poses of selected ligands docked into the HadAB complex with the HadA subunit represented in yellow, the HadB subunit represented in lime green, and the ligand represented in cyan. (A) Compound **12**, the oxadiazolone group occupies the catalytic site mimicking the hydrolytic intermediate with its three H-bond acceptors to the A-Q86 backbone amide, the B-G59 amide, and the catalytic water. (B) Compound **11** forms all the interactions of acyl sulfonamide **9** but with the addition of a H-bond between the B-D36 aspartic acid and the urea NH. (C) Compound **3** makes all the interactions defined with **9** plus the addition of two new H-bonds between the propylamine and A-Q86 and A-E81 at the binding site opening. (D) Compound **15**, lack of activity can be explained by the 5-ethyl substitution to the pyrazole core in a highly polar region clashing with B-D36.

Compound **12** (Figure 5A) with its weak inhibition of *Mtb* HadAB IC_50_ of 4.24 μM, was still able to form H-bonds with backbone amides of A-Q86 and B-G59, but its likely deprotonation (predicted p*K*_a_ of 7.87 ± 0.40 – ACD laboratories) creates a negative charge in the proximity to B-D36. The planarity of the oxadiazolone creates suboptimal geometry for accepting a H-bond from the water and the backbone amide from each subunit. These two factors substantially penalize the binding energy but are not enough to lose all inhibition. Compound **11** (Figure 5B) with a sulfonylurea forms the same S2 interactions as in **9** with the addition of a H-bond between the urea NH and catalytic B-D36, accounting for the potency increase (*Mtb* HadAB IC_50_ = 0.062 μM). Compound **3** (Figure 5C) is another compound with increased potency (*Mtb* HadAB IC_50_ = 0.043 μM) compared to matched-pair **2**. This compound has the same S2 moiety as **9**, but with the addition of the n-propyl amine attached to the 3-phenyl ring interacting with the S3 subsite, a new H-bond and salt bridge are likely formed with A-Y65 and A-E81, respectively. Compound **15** is an example that loses *Mtb* HadAB inhibition significantly with a high IC_50_ of 28 μM despite retaining the acyl sulfonamide pharmacophore and the general core structure of the series. This loss of activity most likely results from a clash of the ethyl group introduced at the 5-position of the central pyrazole core with A-Y65, proximal to the charged B-D36.

These four compounds illustrate the chemical space of *Mtb* HadAB inhibitors from this series, which can be described as a trisubstituted pyrazole with aromatic groups in the 1 and 3 positions and a polar group attached at the 4-position with a 2-atom flexible linker. This polar group needs to be able to accept three H-bonds in an β-unsaturated geometry, mimicking an intermediate of the α–β dehydratase reduction.

The sulfone group in **13** with a methyl oxadiazole linker is torsionally constrained and likely cannot find a low energy conformation where H-bonds with the backbone amides and the water molecule may be formed. This explains the substantial loss in the binding affinity as reflected in the very high HadAB IC_50_.

While HadAB has been identified as the primary target of this series, the HadBC complex also contributes to the fatty acid-ACP dehydratase step of the FAS-II pathway. There is only one published structure of *Mtb* HadBC, in the unliganded state (PDB ID: 5ZY8). Since the catalytic dyad is found on the HadB subunit while the backbone amides are conserved on the HadA and HadC subunits, the S2 catalytic subsite of HadBC remains much the same as the one in HadAB. The significant difference in the compound binding site between these two complexes is the Y65 residue of HadA, which is substituted for K65 in HadC. This tyrosine to lysine substitution causes the HadBC complex binding site to lose much of its aromatic character in the S1 and S3 subsites. This may explain the substantial loss in potency for most of the compounds in the series against HadBC enzyme activity (Table 2, Figure S5). Additionally, as K157R HadC mutation observed in mutants resistant to compound **1** is not positioned in the active site, we speculate that this mutation contributes to resistance via altering HadBC enzyme substrate specificity and activity rather than through changing the affinity to the compounds.

## Conclusion

We showed that 1,3-diarylpyrazolyl-acylsulfonamides identified from a phenotypic screening against *Mtb* target HadAB/BC, 3-hydroxyl-ACP dehydratase complex, an essential component of the FASII pathway in *Mtb.* These compounds inhibit HadAB by noncovalently engaging the catalytic residues of the HadB subunit, mimicking the high energy oxyanion intermediate of the fatty acid-ACP dehydratase reaction. This is a significantly different mode of inhibition to TB pro-drugs ISO and TAC, which are known to covalently modify HadA upon activation by EthA. 1,3-Diarylpyrazolyl-acylsulfonamides and acyl sulfonyl ureas inhibit the HadAB enzymatic activity with potent IC_50_s in the range of 0.02 to 0.13 μM. Some of the compounds were able to inhibit the activity of the HadBC complex as well, albeit with weaker potency–up to 58% inhibition at 50 μM. This is an important finding as the compounds of this series may be developed into dual inhibitors, inhibiting both HadAB and HadBC and avoiding compensation of HadAB inhibition by HadBC. The cocrystal structure of a key compound with *Mtb* HadAB indicated a unique binding mode of the compounds within the HadAB active site pocket. This structural information is of immense value for further drug design and discovery efforts in optimizing this compound class toward delivering a preclinical candidate for TB. Toward these efforts, a docking model based on the HadAB cocrystal structure of the representative compound was developed, which explains the SAR observed in the series and will be instrumental in further designing efforts to optimize the compounds.

## Methods

### Synthesis

The synthesis of most of the compounds in this manuscript is described previously. Please refer to the Supporting Information for the synthesis of compounds **3**, **4**, **6**, **7**, **13**, and **15**.^12^

### Bacterial Strains and Culture Conditions

The avirulent auxotrophic *Mtb* H37Rv strain mc^2^6206 (Δ*panCD*Δ*leuCD*) was grown at 37 °C in Middlebrook 7H9-OADC-0.05% tyloxapol supplemented with 0.2% casamino acids, 48 μg/mL pantothenate, and 50 μg/mL l-leucine. Hygromycin (Hyg; 50 μg/mL) was added as needed.

### MIC Determinations and Spontaneous Resistant Mutant Generation

The susceptibility of *Mtb* H37Rv mc^2^6206 and corresponding *hadABC* overexpressor, *Mtb*/pNIP40b-*hadABC*, to compounds was determined in 96-well microtiter plates at 37 °C in Middlebrook 7H9 base supplemented with 0.4% glucose, 0.2% casamino acids, 48 μg/mL pantothenate, 50 μg/mL l-leucine, 0.08% sodium chloride and 0.05% tyloxapol using the resazurin blue test. Cultures of wild-type *Mtb* were grown at 37 °C to an OD_600_ = 0.8, pelleted by centrifugation, and resuspended in Middlebrook 7H9 broth supplemented with glycerol, OADC, and 0.05% Tween 80. Aliquots containing 10^9^ cells were plated on standard Middlebrook 7H10 agar plates in the presence of compounds at 5×, 10×, or 20× the MIC_90_ value determined in liquid culture. Colonies arising after 4–5 weeks’ incubation were picked and subcultured in Middlebrook 7H9 broth, and the resistance phenotype was tested.^22^

### Whole-Cell Radiolabeling Experiments

Metabolic labeling of wild-type *Mtb* H37Rv mc^2^6206 with [1,2-^14^C]acetic acid (0.5 μCi/mL; specific activity, 52 Ci/mol, PerkinElmer) was performed for 24 h at 37 °C with shaking. [1,2-^14^C]acetic acid was added to the cultures simultaneously as the compounds. The preparation of fatty acid and mycolic acid methyl esters from whole cells followed earlier procedures.^10^ [1,2-^14^C]acetic acid-derived fatty acid and mycolic acid methyl esters were separated by TLC on aluminum-backed silica gel 60-precoated plates F254 (E. Merck) and revealed by PhosphorImaging.

### Dehydratase Assays

HadAB and HadBC were produced and purified from *E. coli*.^14,15^ The enzymatic activity of HadAB (22 nM) and HadBC (130 nM) was measured in the presence of *trans*-2-hexadecenoyl-CoA (*trans*-2-C_16:1_-CoA) (60 μM in the HadAB assay; 25 μM in the HadBC assay) using the spectrophotometric assay described previously.^14^ The IC_50_ values were obtained with *GraFit* (Version 5.0.13) using the following equation:
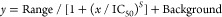
where Range is the fitted uninhibited value minus the Background and *s* is a slope factor.

### Chemoproteomics

Chemoproteomics experiments were performed as previously described.^16^ Briefly, NHS-activated sepharose beads were derivatized with compounds **3** or **4** at a concentration of 0.5 or 1 mM and washed and equilibrated in lysis buffer (50 mM Tris-HCl, pH 7.4, 0.4% Igepal-CA630, 1.5 mM MgCl_2_, 5% Glycerol, 150 mM NaCl, 25 mM NaF, 1 mM Na_3_VO_4_, 1 mM DTT, and one Complete EDTA-free protease inhibitor tablet (Roche) *per* 25 mL). The functionalized beads were incubated at 4 °C for 1 h with 0.1 mL (0.25 mg) *M. bovis* BCG extract, preincubated with a test compound or DMSO (vehicle control). *M. bovis* BCG extract was generated as described previously.^23^ The experimental setup was such that 10 samples were measured in parallel (TMT 10-plex)^24^ to generate values for the affinity of the beads to the bound proteins (“depletion” values, four samples) and to generate IC_50_ values (six samples) in a single experiment. Samples 1 and 2 were the vehicle control, samples 3 and 4 were done in the same way, but while the beads were discarded after the first incubation step, the extract was incubated with fresh beads to measure how much protein could rebind to the fresh beads (the protein was depleted from the extract by the first bead-binding).^17^ Apparent dissociation constants were determined by considering the protein depletion by the beads.^16^ Samples 5–10 were used to generate IC_50_ values by adding compounds over a range of concentrations (40 μM, 1:3 dilutions). To analyze the correlation between target binding and antimycobacterial activity, we performed experiments as described above, but the compounds for the competition were added at a single concentration of 10 μM and compared to a vehicle control (in replicate). Beads were transferred to Filter plates (Durapore (PVDF membrane, Merck Millipore), washed extensively with lysis buffer, and eluted with SDS sample buffer. Proteins were digested with trypsin following a modified single pot solid-phase sample preparation (SP3) protocol.^25,26^ Peptides were labeled with isobaric mass tags (TMT10, Thermo FisherScientific, Waltham, MA) using the 10-plex TMT reagents,^24,27^ and labeled peptide extracts were combined to a single sample per experiment, lyophilized and subjected to LC-MS analysis.^28,29^ LC-MS/MS measurements using Q Exactive Orbitrap or Orbitrap Fusion Lumos mass spectrometers (Thermo Fisher Scientific) were performed as described.^28,30^ Unless stated otherwise, we accepted protein identifications as follows: (i) For single-spectrum to sequence assignments, we required this assignment to be the best match and a minimum Mascot score of 31 and a 10× difference of this assignment over the next best assignment. Based on these criteria, the decoy search results indicated a less than 1% false discovery rate (FDR). (ii) For multiple spectra to sequence assignments and using the same parameters, the decoy search results indicated less than 0.1% FDR. Quantified proteins were required to contain at least two unique peptide matches. FDR for quantified proteins was less than 0.1%. Raw data tables for the chemoproteomics experiments can be found in Tables S1 and S2.

### Cocrystallization, Data Collection, and Crystal Structure Determination

HadAB complex for crystallization was purified from the coexpression construct as previously described.^14^ For additional purity of the sample, the gel filtration chromatography step on the Sephadex S75 column was added. Purified protein in 50 mM TRIS-HCl pH 7.5 with 150 mM NaCl, 10% glycerol, and 1 mM DTT was concentrated to 6 mg/mL and mixed with 0.5 mM final concentration of the inhibitor at 1% final DMSO. Crystals were obtained by sitting drop vapor diffusion method after mixing equal volumes of protein-inhibitor solution and mother liquor (0.1 M Citric Acid: NaOH pH 3.5, 25% PEG3350). The crystal was cryo-protected before flash freezing by briefly soaking it in 25% ethylene glycol in the mother liquor. Diffraction data were collected at APS synchrotron, beamline 23IDD. The data were initially processed by XDS,^31^ followed by indexing and scaling in POINTLESS^32^ and AIMLESS.^33^ The structure was solved by molecular replacement with MOLREP using 4rlw as a search model.^34^ The initial model was improved through the iterative rounds of manual building in COOT^35^ and refinement in PHENIX.^36^ Data scaling and refinement statistics can be found in Table S4.

## Supporting Information Available

Screening method and profiling of selected compounds against ESKAPE pathogens; phenotypic and genotypic profiling of compound **1**-resistant mutants; structures of compounds utilized to perform chemoproteomics experiment; chemoproteomic profiling using a **3**-bead matrix; scatter plot of HadAB pIC_50_ vs *Mtb* pMIC; profiling of 1,3-diarylpyrazolyl-acylsulfonamides against *Mtb* H37Rv mc^2^6206 WT and overexpressing *hadABC* strains; crystallography data collection and refinement statistics, pdb ID; synthetic pathways and analytical data for compounds **3**, **4**, **6**, **7**, **13**, and **15**; ligand density map of the bound inhibitor **9** in complex with *Mtb* HadAB; 3D illustration of the binding modes of butein and fisetin in the HadAB complex; unliganded structure of the *Mtb*HadBC superimposed onto the crystal structure of *Mtb*HadAB in complex with **9** (PDF)
