# Supplementary material for: 1,3-Diarylpyrazolyl-acylsulfonamides Target HadAB/BC Complex in Mycobacterium tuberculosis
Source: ACS Infect Dis. 2022 Nov 3;8(11):2315–26. doi: 10.1021/acsinfecdis.2c00392 (PMC9673142; doi:10.1021/acsinfecdis.2c00392)
Supplement: Supplementary file 1 — id2c00392_si_001.pdf [file id2c00392_si_001.pdf]

Supplementary Information

1,3-Diarylpyrazolyl-acylsulfonamides target HadAB/BC complex in *Mycobacterium tuberculosis*

Vinayak Singh,[a,b] Anna E. Grzegorzewicz,[c] Stephen Fienberg,[a] Rudolf Müller,[a] Lutete Peguy Khonde,[a] Olalla Sanz,[d] Salvatore Alfonso,[d] Beatriz Urones,[d] Gerard Drewes,[e] Marcus Bantscheff,[e] Sonja Ghidelli-Disse,[e] Thomas R. Ioerger,[f] Bhanupriya Angala,[c] Jiuyu Liu,[g] Richard E. Lee,[g] James C. Sacchettini,[h] Inna V. Krieger,*[h] Mary Jackson,*[c] Kelly Chibale,*[a,b] Sandeep R. Ghorpade*[a]

[a]Drug Discovery and Development Centre (H3D), University of Cape Town, Rondebosch 7701, South Africa; [b]South African Medical Research Council Drug Discovery and Development Research Unit, Department of Chemistry and Institute of Infectious Disease and Molecular Medicine, University of Cape Town, Rondebosch 7701, South Africa; [c]Mycobacteria Research Laboratories, Department of Microbiology, Immunology and Pathology, Colorado State University, Fort Collins, Colorado 80523-1682, USA; [d]Global Health Pharma Research Unit, GlaxoSmithKline, Severo Ochoa 2, Tres Cantos, 28760, Madrid, Spain; [e]Cellzome GmbH · A GSK Company, Meyerhofstrasse 1, 69117 Heidelberg, Germany; [f]Department of Computer Science and Engineering, Texas A&M University, College Station 77843 Texas, United States; [g]Department of Chemical Biology & Therapeutics, St. Jude Children's Research Hospital, 262 Danny Thomas Place, Memphis, TN 38105, USA; [h]Texas A&M University, Department of Biochemistry and Biophysics, ILSB 2138, 301 Old Main Dr, College Station, TX 77843-3474, USA;

*Corresponding Authors

E-mail: krigin@gmail.com; Mary.Jackson@colostate.edu; Kelly.Chibale@uct.ac.za; Sandeep.Ghorpade@uct.ac.za

Contents:

**Methods**. ESKAPE Antimicrobial Screening	S3
**Table S1**. Profiling of selected compounds against ESKAPE pathogens.	S3
**Table S2**. Phenotypic and genotypic profiling of compound **1**-resistant mutants	S4
**Table S3**. Structures of compounds utilized to perform chemoproteomics profiling experiments	S5
**Figure S1**. Chemoproteomic profiling using **3**-bead-matrix	S6
**Figure S2**. Scatter plot of HadAB pIC_{50} vs *Mtb* pMIC	S7
**Table S4**. 1,3-diarylpyrazolyl-acylsulfonamides against *Mtb* H37Rv $mc^2$6206 WT and overexpressing *hadABC* strains.	S8
**Table S5**. Crystallography Data collection and refinement statistics, pdb ID	S9
**Chemistry**. Synthesis and analytical data for compounds **3**, **4**, **6**, **7**, **13** and **15**	S10
**Figure S3**. Ligand Density map of the bound inhibitor **9** in Complex with Mtb HadAB	S29
**Figure S4**. A 3D illustration of the binding modes of butein and fisetin in the HadAB complex - PDB ID: 4RLW and 4RLT respectively	S30
**Figure S5**. The unliganded structure of the *Mtb*HadBC superimposed onto the crystal structure of *Mtb*HadAB in complex with **9** (turquoise).	S31
**References**	S32

Methods: ***ESKAPE Antimicrobial Screening***

The selectivity of compounds was assessed in a microdilution broth method against the selected Gram negative and one Gram positive strains, in accordance with the Clinical and Laboratory Standards Institute guidelines (CLSI, 2017). The bacterial strains used in this assay included *Escherichia coli* ATCC 25922, *Acinetobacter baumannii* ATCC 19606, *Staphylococcus aureus* ATCC 25923, *Pseudomonas aeruginosa* ATCC 27853, and *Klebsiella pneumoniae* ATCC BAA-1705, and *Enterobacter cloacae* ATCC 700323. Overnight cultures of the different bacterial strains were grown on Müller-Hinton (MH) agar plates at 37˚C. Inoculum cultures were prepared in phosphate buffer saline (PBS) to a 0.5 McFarland (OD600 = 0.08) and diluted in MH broth to yield a bacterial concentration of approximately 1.5 x 10^6 cfu/mL. The compounds were tested at a concentration range between 0.25 – 128 µM. Furthermore, three antibiotics (ciprofloxacin, gentamycin, moxifloxacin, and kanamycin) were used as controls. Untreated bacterial controls as well as media sterility controls were included in the assay. The prepared plates were incubated at 37°C for 16 hours, followed by a visual assessment. The minimum inhibitory concentration (MIC) values were determined as the lowest concentration where no bacterial growth could be observed.

Table S1. Profiling of selected compounds against ESKAPE pathogens.

	Escherchia coli (ATCC 25922)	*Staphylococcus aureus* (ATCC 25923)	*Klebsiella pneumoniae* (ATCC BAA-1705)	*Acinetobacter baumannii* (ATCC 19606)	*Pseudomonas aeruginosa* (ATCC 27853)	*Enterobacter cloacae* (ATCC 700323
Compound	MIC (µM)					
**1**	>128	>128	>128	>128	>128	>128
**2**	>128	128	>128	>128	>128	>128
**11**	>128	>128	>128	>128	>128	>128
**14**	128	>128	>128	>128	>128	>128
Ciprofloxacin	0.06	0.5	>32	1	0.125	0.06
Gentamycin	2	0.5	4	16	2	2
Moxifloxacin	0.06	0.06	>32	0.5	2	0.06
Kanamycin	4	2	>32	16	>32	8

Table S2. Phenotypic and genotypic profiling of compound 1-resistant mutants

Strain	Compound 1 MIC (µM)	Mutation	Comment
H37RvMA	1.9	-	WT reference strain
SRM59-1	50-100	Rv0637/*hadC*:K157R<br>Rv2932/*ppsB*:T1300N	Both *hadC* and *ppsB* are *in vitro* non-essential genes.
SRM59-2	50-100	Rv0637/*hadC*:K157R<br>Rv2932/*ppsB*:T1300N	
SRM59-3	50-100	Rv0637/*hadC*:K157R<br>Rv2932/*ppsB*:T1300N	

Table S3. Compound names and structures utilized to perform chemoproteomic profiling experiments.

Compound	Structure	*Mtb* pMIC99
**1**	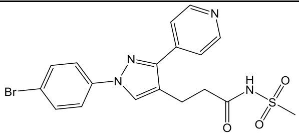	6.2
**2**	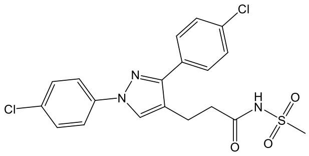	5.5
**3**	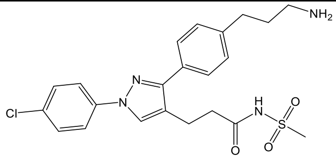	6
**4**	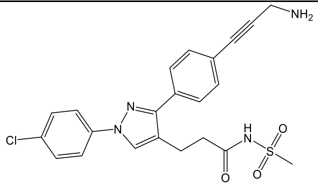	5.4
**5**	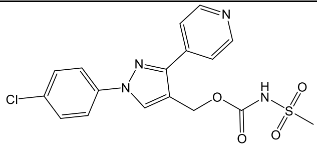	5.6
**6**	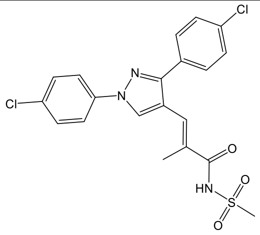	<4.3
**7**	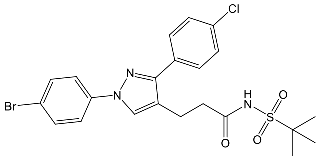	<4.3
**8**	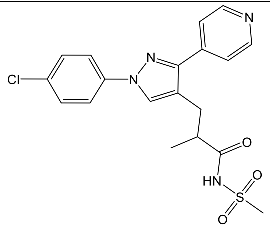	<4.3

Mtb pMIC defined as $-\log_{10}(\mathrm{MIC}_{99}\text{ in M})$

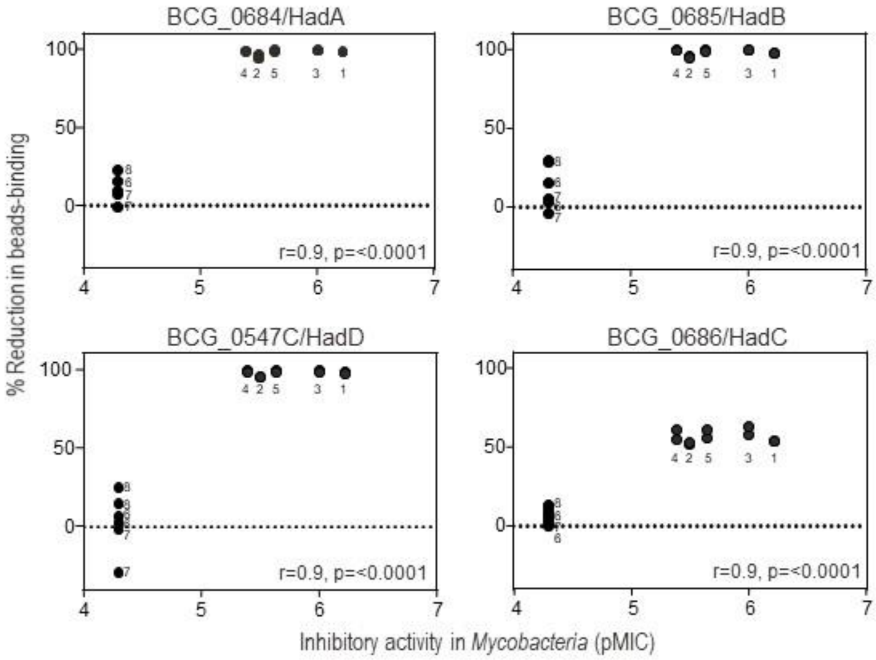

Compound	pMIC99
1	6.2
2	5.5
3	6.0
4	5.4
5	5.6
6	4.3
7	4.3
8	4.3

Figure S1. Chemoproteomic profiling using **3**-bead-matrix

Chemoproteomic profiling showed only a weak effect on HadC by 1,3-Diarylpyrazolyl-acylsulfonamide series compounds. Correlation of target binding and anti-mycobacterial activity for compounds from the 1,3-Diarylpyrazolyl-acylsulfonamide series. Experiments were performed as described in **Fig 1**. 5 compounds from the series with activity against *Mtb* and 3 that did not show activity (see table) were analyzed at 10 µM in two independent replicates on **3**-bead-matrix. Active compounds showed inhibition of proteins HadA, HadB, BCG_0547c (HadD) and HadC from **3**-bead-binding, while inactive compounds did not show any. HadA/B/D inhibition from bead-binding is in the range of 94-100%, while for HadC the inhibition is in a range of 52-64%, which indicates weaker potential IC_{50}-values for HadC. r: Pearson correlation coefficient; p: p-value (calculated probability), *Mtb* pMIC defined as $-\log_{10}(MIC_{99}$ in M).

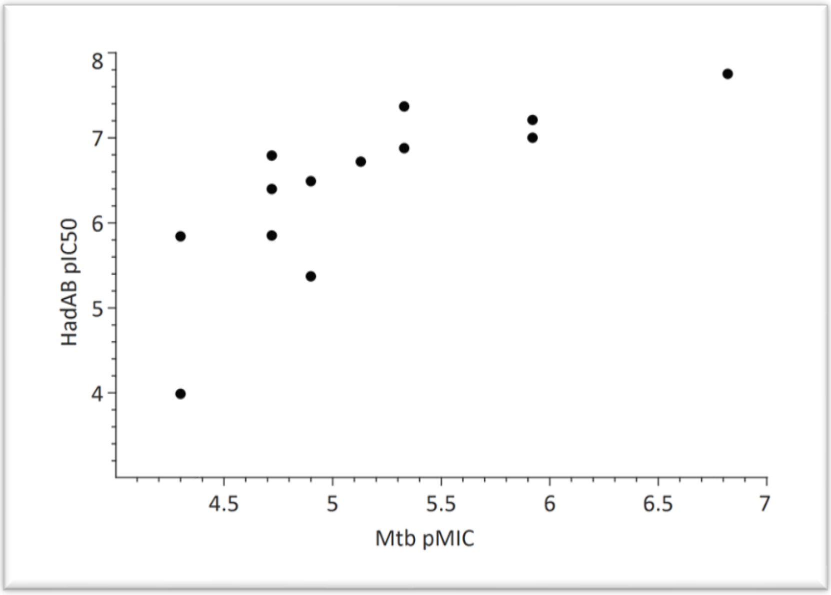

Figure S2. Scatter plot of HadAB pIC_{50} [defined as -log10(HadAB IC_{50} in Molar units)] vs *Mtb* pMIC [defined as $-\log_{10}$(MIC in M)]

Table S4. 1,3-diarylpyrazolyl-acylsulfonamides against *Mtb* H37Rv mc^{2}6206 WT and overexpressing *hadABC* strains.

Compound	*Mtb* WT	*Mtb*/pNIP40b-*hadABC*
**2**	<0.625	2.5
**3**	5	20
**10**	1.5	3
**11**	0.625	>40
**12**	3.125	12.5
**13**	3.75	1.9
**14**	<0.243	15
**15**	3.9	7.8

MICs were determined in 96-well microtiter plates in Middlebrook 7H9 broth supplemented with 0.4% glucose, 0.2% casamino acids, 48 µg/mL pantothenate, 50 µg/mL L-leucine, 0.08% sodium chloride and 0.05% tyloxapol using resazurin blue test (REMA). MICs are expressed in µM.

Table S5. Crystallography Data collection and refinement statistics, pdb ID 7SVT

**Data collection**	
Space group	$P2_12_12_1$
Cell dimensions	
a, b, c (Å)	85.3, 107.2, 142.07
α, β, γ (°)	90.00, 90.00, 90.00
Resolution (Å)	48.6 – 2.4 (2.47-2.4)
R_{sym} or R_{merge}	0.198 (1.962)
$I/\sigma I$	7.5 (1.2)
Completeness (%)	99.9 (100)
Redundancy<br>CC1/2	5.8 (6)<br>0.994 (0.536)
**Refinement**	
Resolution (Å)	45.38 – 2.4
No. reflections	51470
R_{work}/ R_{free}	0.2173 / 0.2751
No. atoms	
Protein	8976
Ligand/ion(s)	140
Water	88
B-factors	
Protein	52
Ligand/ion(s)	46.8
R.m.s deviations	
Bond lengths (Å)	0.01
Bond angles (º)	1.232

*Highest resolution shell is shown in parenthesis.

Chemistry

Unless otherwise stated, the starting materials, reagents and solvents were purchased as high-grade commercial products. Analytical TLC was performed on Merck silica gel (60F254) precoated plates (0.25 mm). The compounds were visualized under UV light (254 nm) and/or stained with the relevant reagent. Flash column chromatography was performed on silica gel with pore size 60 Å, 230−400 mesh particle size, and 40−63 μm particle size, with the indicated solvents. The yields refer to the purified products, and they were not optimized. All the solid compounds were obtained as amorphous solids, and melting points were not measured. ^{1}H NMR spectra were recorded on a Bruker Avance III NMR spectrometer and in a Bruker DPX Avance 400 MHz instrument equipped with a QNP probe and are reported in ppm using tetramethylsilane as internal standard. Mass spectra data measurements were performed on a VG-Analytical Autospec Q mass spectrometer. Analytical purity was ≥95% unless stated otherwise. The purities of the final compounds were determined by using a Waters ZQ2000 coupled with LC Waters 2795 and Waters 2996 PDA detector.

High-resolution mass method:

HPLC–HRMS was obtained using an AB Sciex® X500R QTOF coupled to an AB Sciex® Exion LC system. Spectral data were obtained using information dependent acquisition (IDA) at a mass range of 50–1200 Da. All methods, batches and data were processed using OS Sciex® v1.2. The declustering potential was 80 V, the curtain gas (N_2) was at 25 pounds per square inch (psi), the ion spray voltage was 5500 V, and the source temperature was 450°C. Ion source gases 1 and 2 were at 45 and 55 psi, respectively. The collision energy was 10 eV for the MS scans and 20–50 eV for MS/MS scans. The IDA intensity threshold was 50 cycles per second. The aqueous mobile phase used was 1 mM ammonium formate in water, and the organic mobile phase was methanol with 0.5% formic acid. The flow rate was 700 µL/minute, and the method ran from 2% to 98% organic for 25 minutes, was held at 98% organic for a further 2 minutes before returning to 2% organic over 3 minutes to equilibrate for the next run. A Kinetex® C18 LC column (5 µm, 100 Å, 150 mm × 4.6 mm) with a column protector was used. A blank injection was run between each sample. The spectra were processed and displayed using MZMine2.5.[1]

Scheme S1. Synthetic pathway for compounds 3 and 4.

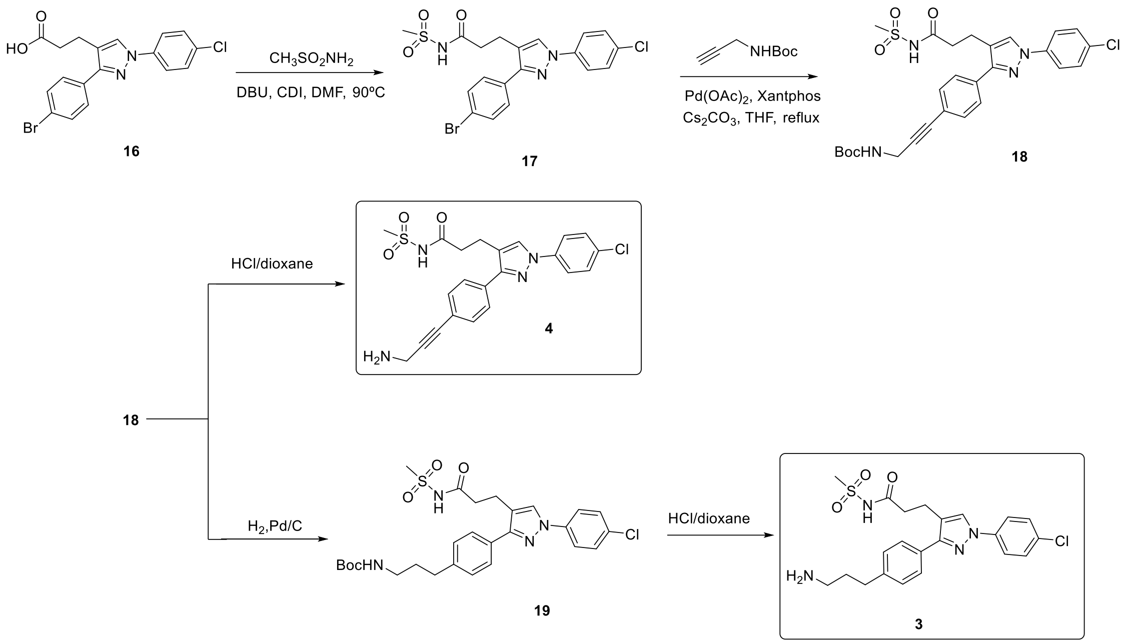

3-(3-(4-bromophenyl)-1-(4-chlorophenyl)-1*H*-pyrazol-4-yl)-*N*-(methylsulfonyl) propanamide (17). To a stirring solution of **16** (500 mg, 1.233 mmol) in DMF (5 mL), CDI (400 mg, 2.465 mmol) was added and the solution was stirred at room temperature for 10 min. Methanesulfonamide (141 mg, 1.479 mmol) and DBU (0.223 mL, 1.479 mmol) were added and the reaction mixture stirred overnight at 90 °C. Solvent was removed under reduced pressure and the crude obtained was partitioned between ethyl acetate and NH_4Cl sat. The organic layer was washed with brine, dried over magnesium sulphate, filtered and concentrated under reduced pressure. The crude obtained was purified by silica gel column chromatography (eluents DCM/MeOH, from 0 to 2% of MeOH), to yield **17** as a yellow solid (32% yield). ^{1}H NMR (400 MHz, DMSO-d_6) δ 11.73 (s, 1H), 8.42 (s, 1H), 7.92 – 7.84 (m, 2H), 7.68 (s, 4H), 7.61 – 7.54 (m, 2H), 3.20 (s, 3H), 2.96 – 2.87 (m, 2H), 2.62 (t, J = 7.5 Hz, 2H).

***Tert*-butyl (3-(4-(1-(4-chlorophenyl)-4-(3-(methylsulfonamido)-3-oxopropyl)-1*H*-pyrazol-3-yl)phenyl)prop-2-yn-1-yl)carbamate (18)**.Under nitrogen atmosphere, **17** (100 mg, 0.207 mmol), *tert*-butyl prop-2-yn-1-ylcarbamate (48.2 mg, 0.311 mmol), Palladium(II) acetate (4.65 mg, 0.021 mmol), xantphos (5.99 mg, 10.36 µmol), and cesium carbonate (202 mg, 0.621 mmol) were placed in THF (2.5 mL). The mixture was heated to reflux overnight. The reaction solvent was evaporated under reduced pressure and HCl 2N was added (15 mL). The mixture was extracted with ethyl acetate (20 mL), washed with brine (20 mL), filtered through a celite cartridge and dried over sodium sulphate. The crude product was purified by silica gel column chromatography (DCM:MeOH 99:1; isocratic method). The product fractions were collected and purified again by preparative HPLC (XBrigde C18, X_BRIDGE 19x150 60_100 ACID 0.1% TFA in water/ 0.1% TFA in ACN affording **18** as a white solid (12% yield). ^{1}H NMR (400

MHz, $CDCl_3$) δ 8.07 (br s, 1H), 7.74 (s, 1H), 7.62 – 7.53 (m, 4H), 7.42 (d, *J* = 8.6 Hz, 2H), 7.34 (d, *J* = 9.1 Hz, 2H), 4.75 (br t, *J* = 5.1 Hz, 1H), 4.22 – 3.99 (m, 2H), 3.18 (s, 3H), 3.01 (t, *J* = 7.2 Hz, 2H), 2.50 (t, *J* = 7.3 Hz, 2H), 1.41 (s, 9H). MS-ES$^+$ $[M+H]^+$ = 557

3-(3-(4-(3-aminoprop-1-yn-1-yl)phenyl)-1-(4-chlorophenyl)-1*H*-pyrazol-4-yl)-*N*-(methylsulfonyl)propenamide (4). To a solution of **18** (34 mg, 0.061 mmol) in 1,4-dioxane (1.526 mL), HCl (4M in 1,4 dioxane) (1.526 mL, 6.10 mmol) was added and the reaction mixture was stirred at rt for 4 h. The reaction mixture was evaporated under reduced pressure. The crude obtained was dissolved in MeOH and evaporated. This process was repeated several times (to remove the excess of acid). The solid obtained was dissolved in MeOH and passed through a SCX (1g cartridge) and after eluting with MeOH three column volumes, desired product was eluted with ammonia in methanol. Solvent was evaporated to yield **4** as pale brown solid (78% yield). ^{1}H NMR (400 MHz, DMSO-*d*$_6$) δ 8.42 (s, 1H), 7.89 (d, *J* = 8.8 Hz, 2H), 7.80 (d, *J* = 8.6 Hz, 2H), 7.59 – 7.54 (m, 5H), 3.91 (s, 2H), 2.86 (s, 3H), 2.89 – 2.82 (m, 1H), 2.40 (dd, *J* = 8.7, 6.9 Hz, 2H). MS-ES$^+$ $[M+H]^+$ = 457; HPLC purity >95%.

***Tert*-butyl (3-(4-(1-(4-chlorophenyl)-4-(3-(methylsulfonamido)-3-oxopropyl)-1*H*-pyrazol-3-yl)phenyl)propyl)carbamate (19)**. **18** (56 mg, 0.101 mmol) was dissolved in 40 mL of MeOH/DCM (1:1) and it was hydrogenated in the H-Cube PRO through a catalyst cartridge of palladium on carbon 10% (Pd/C 10% "CatCart") under the following conditions: full H_2, flow: 1 mL/min, temperature: room temperature, pressure: 1 bar and the reaction was passed through the cartridge for 40 min. Solvent was concentrated under reduced pressure and the crude purified by SFC, to yield **2** as white solid (25% yield). ^{1}H NMR (400 MHz, DMSO-*d*$_6$) δ 11.74 (s, 1H), 8.38 (s, 1H), 7.87 (d, *J* = 9.1 Hz, 2H), 7.62 (d, *J* = 8.3 Hz, 2H), 7.57 (d, *J* = 9.1 Hz, 2H), 7.31 (d, *J* = 8.1 Hz, 2H), 6.88 (br t, *J* = 5.2 Hz, 1H), 3.21 (s, 3H), 3.01 – 2.86 (m, 4H), 2.66 – 2.57 (m, 4H), 1.71 (quin, *J* = 7.3 Hz, 2H), 1.38 (s, 9H). MS-ES$^+$ $[M+H]^+$ = 561

3-(3-(4-(3-aminopropyl)phenyl)-1-(4-chlorophenyl)-1*H*-pyrazol-4-yl)-*N*-(methylsulfonyl)propenamide (3). To a solution of **19** (103 mg, 0.184 mmol) in 1,4-dioxane (4.59 mL), was added HCl (4M in 1,4 dioxane) (4.59 mL, 18.36 mmol) and the reaction mixture was stirred at rt for 3 h. The reaction mixture was evaporated under reduced pressure. The crude obtained was dissolved in MeOH and evaporated. This process was repeated several times (to remove the excess of acid). The solid obtained was dissolved in MeOH and passed through a SCX (1g cartridge) and after eluting with MeOH three column volumes, desired product was eluted with ammonia in methanol. Solvent was evaporated to yield **3** as pale brown solid (73% yield). ^{1}H NMR (400 MHz, DMSO-*d*$_6$) δ 8.38 (s, 1H), 7.87 (d, *J* = 9.1 Hz, 2H), 7.66 (d, *J* = 8.1 Hz, 3H), 7.55 (d, *J* = 8.8 Hz, 2H), 7.33 (d, *J* = 8.3 Hz, 2H), 2.84 – 2.76 (m, 4H), 2.74 (s, 3H), 2.70 (t, *J* = 7.6 Hz, 2H), 2.32 – 2.25 (m, 2H), 1.88 (quin, *J* = 7.6 Hz, 2H). ^{13}C NMR (151 MHz, MeOD) δ 186.1, 160.0, 149.8, 147.9, 140.7, 139.2, 138.8 (2C), 137.9 (2C), 137.0 (2C), 137.0, 131.2, 128.8 (2C), 49.7, 49.5, 47.9, 41.0, 38.1, 30.5. HRMS: calculated for $C_{22}H_{26}ClN_4O_3S$ $[M+H]^+$, 461.1409 found 461.1429. HPLC purity >95%.

^{1}H NMR spectrum of compound **4**

^{1}H NMR (400 MHz, DMSO-d_6) δ 8.42 (s, 1H), 7.89 (d, J = 8.8 Hz, 2H), 7.80 (d, J = 8.6 Hz, 2H), 7.59 – 7.54 (m, 5H), 3.91 (s, 2H), 2.86 (s, 3H), 2.89 – 2.82 (m, 1H), 2.40 (dd, J = 8.7, 6.9 Hz, 2H)

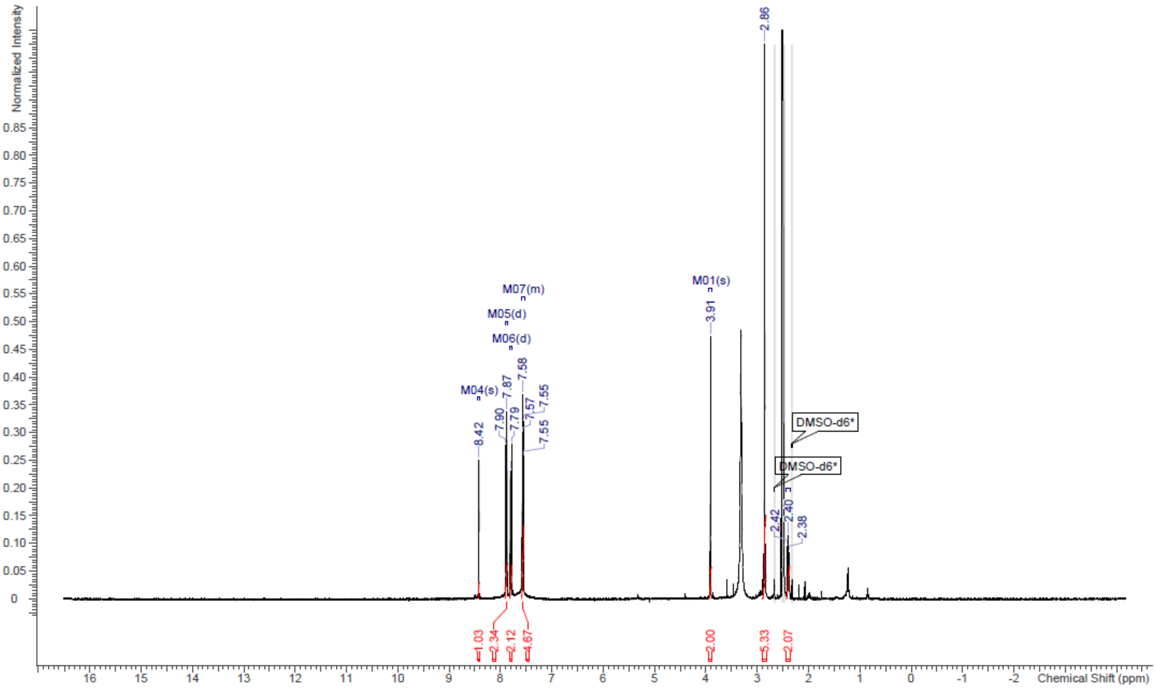

LC-MS chromatogram of compound **4**

Sample amount: 1 mg
Solvent: DMSO
Concentration: 1 mg/mL
Column: Acquity UPLC BEH C18 1.7u 3x50mm
Method: 0.1% Formic Acid in Water / 0.1% Formic Acid in ACN
Initial 95:5
0.0 – 1.4 min 0:100
1.4 – 1.9 min 0:100
1.9 – 2.0 min 95:5
Flow: 0.8 mL/min
Inj. Vol.: 0.2 uL
Tª: 50ºC

Retention Time(min)	Molecular Formula	Monoisotopic Mass	$[M+H]^+$	$[M-H]^-$	Purity (297nm)
0.91	C22H21ClN4O3S	456.10	**457**		>95

Chromatograms & Spectra:

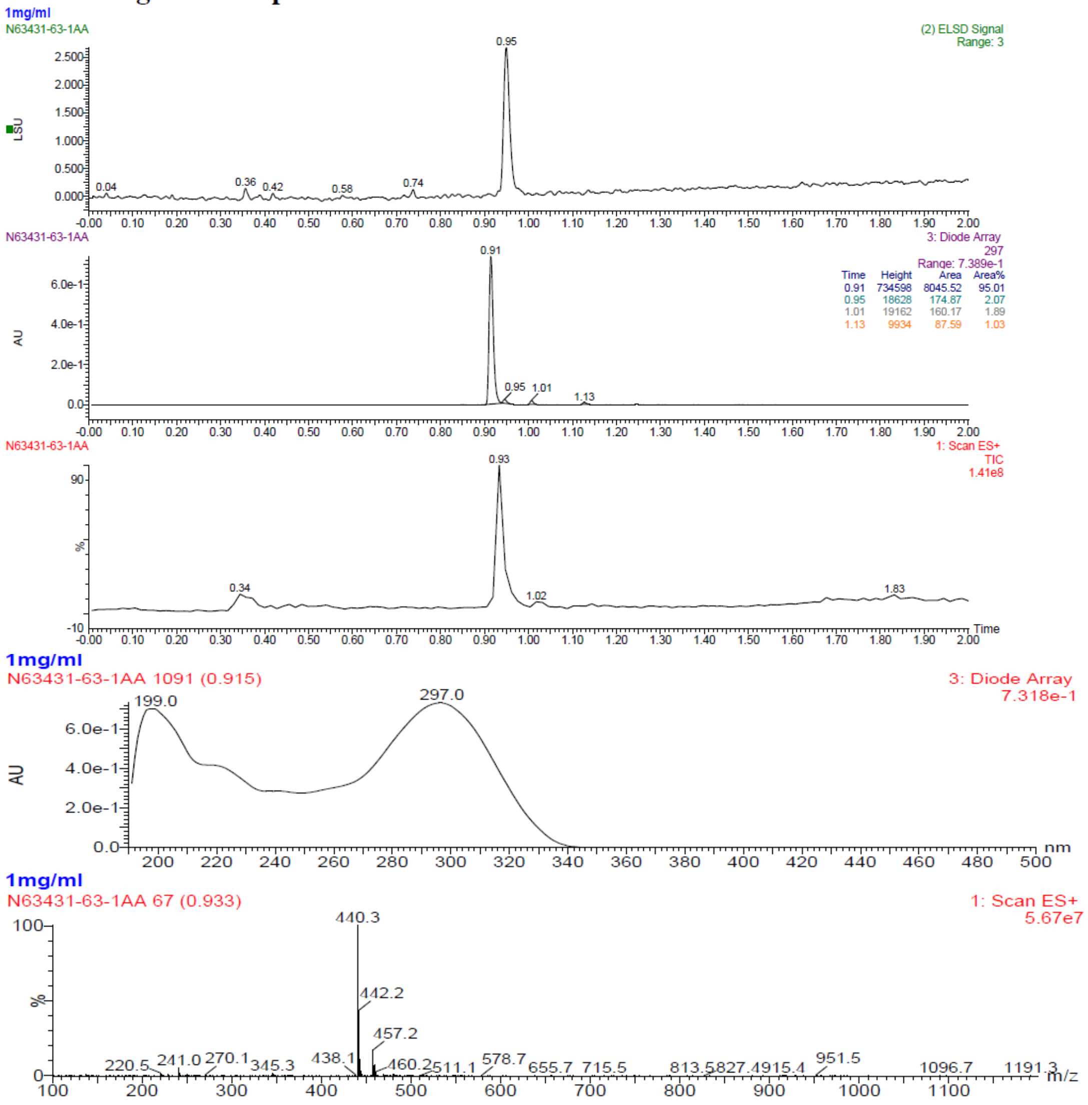

^{1}H NMR spectrum of compound **3**

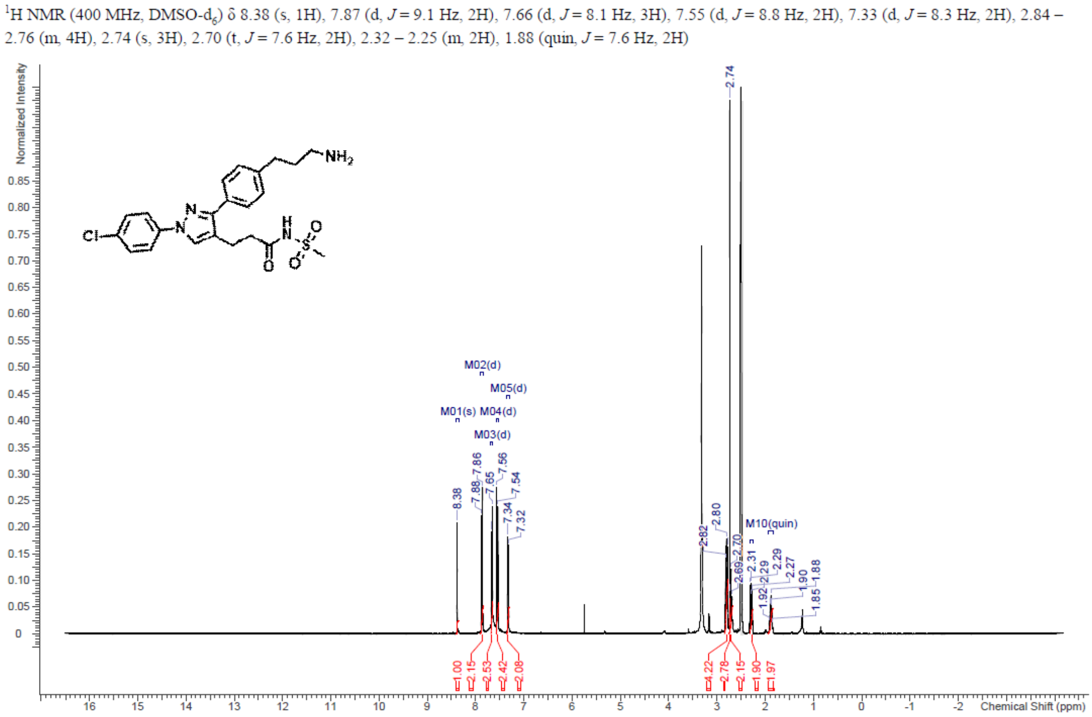

^{13}C NMR spectrum of compound **3**

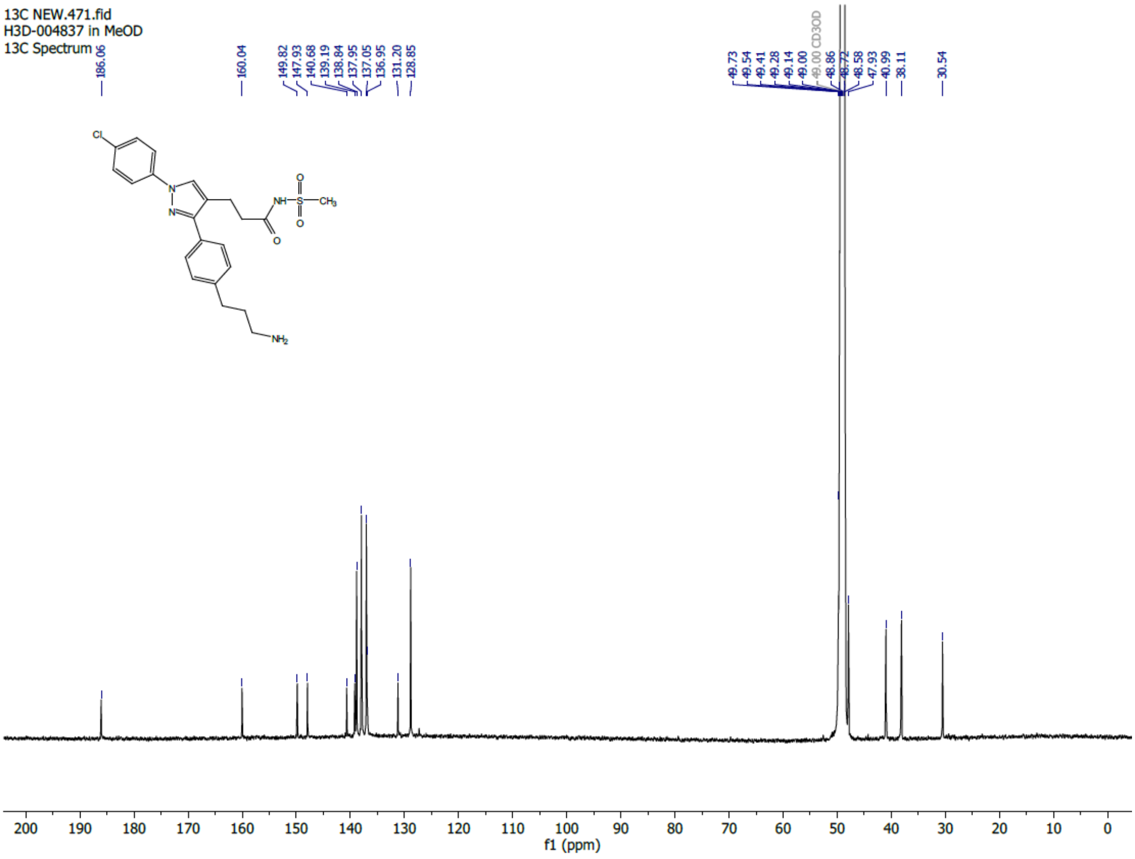

LC chromatogram of compound **3**

Sample amount: 1 mg
Solvent: DMSO
Concentration: 1 mg/mL
Column: Acquity UPLC BEH C18 1.7u 3x50mm
Method: 0.1% Formic Acid in Water / 0.1% Formic Acid in ACN
Initial 95:5
0.0 – 1.4 min 0:100
1.4 – 1.9 min 0:100
1.9 – 2.0 min 95:5
Flow: 0.8 mL/min
Inj. Vol.: 0.2 uL
Tª: 50ºC

Retention Time(min)	Molecular Formula	Monoisotopic Mass	$[M+H]^+$	$[M-H]^-$	Purity (287nm)
0.91	C22H25ClN4O3S	460.13	**461**		>95

Chromatograms & Spectra:

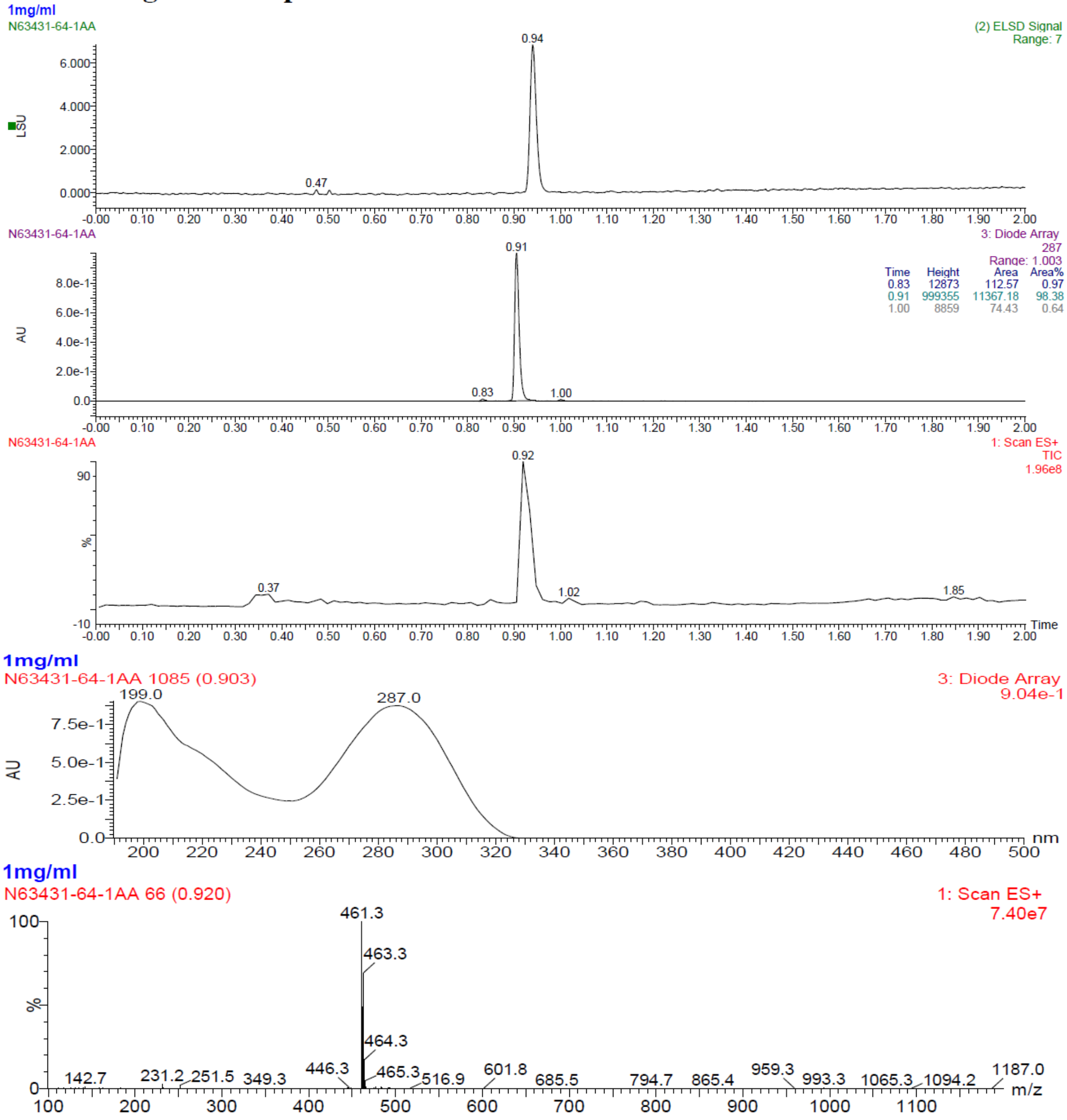

Synthesis of (*E*)-3-(1,3-*bis*(4-chlorophenyl)-1*H*-pyrazol-4-yl)-2-methyl-*N*-(methylsulfonyl)acrylamide (6)

Compound **6** was synthesized as described for compound **17** and reported earlier.[2] Compound **6** was isolated as a white crystal. Yield: 19%. ^{1}H NMR (300 MHz, DMSO-d_6) δ 11.66 (s, 1H), 8.84 (s, 1H), 8.11 – 8.00 (m, 2H), 7.76 – 7.67 (m, 2H), 7.68 – 7.55 (m, 4H), 7.33 (s,1H), 2.12 (d, J = 1.2 Hz, 3H). ^{13}C NMR (151 MHz, DMSO-d_6) δ 168.3, 151.7, 137.8, 133.5, 131.2, 130.8, 130.2 (2C), 129.5 (2C), 129.3, 129.1, 128.8 (2C), 127.3, 120.6 (2C), 116.4, 41.2, 14.5. HRMS: calculated for $C_{20}H_{18}Cl_2N_3O_3S$ [M+H]$^+$, 450.0440 found 450.0453. HPLC purity 97%.

^{1}H NMR spectrum of compound **6**

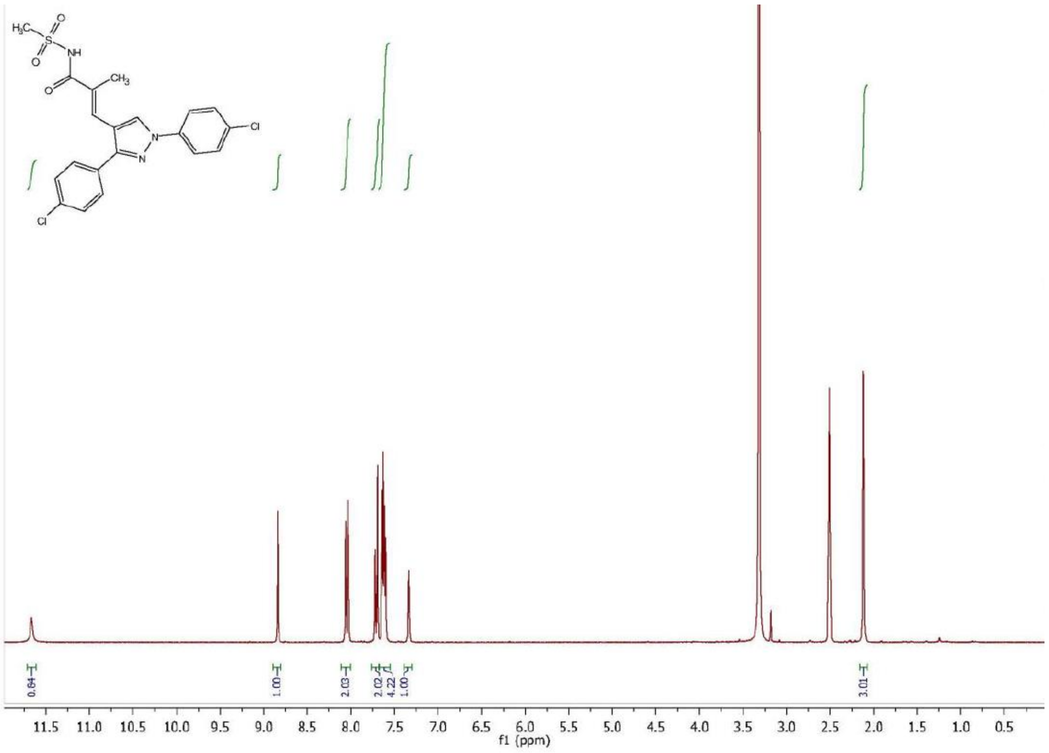

^{13}C NMR spectrum of compound **6**

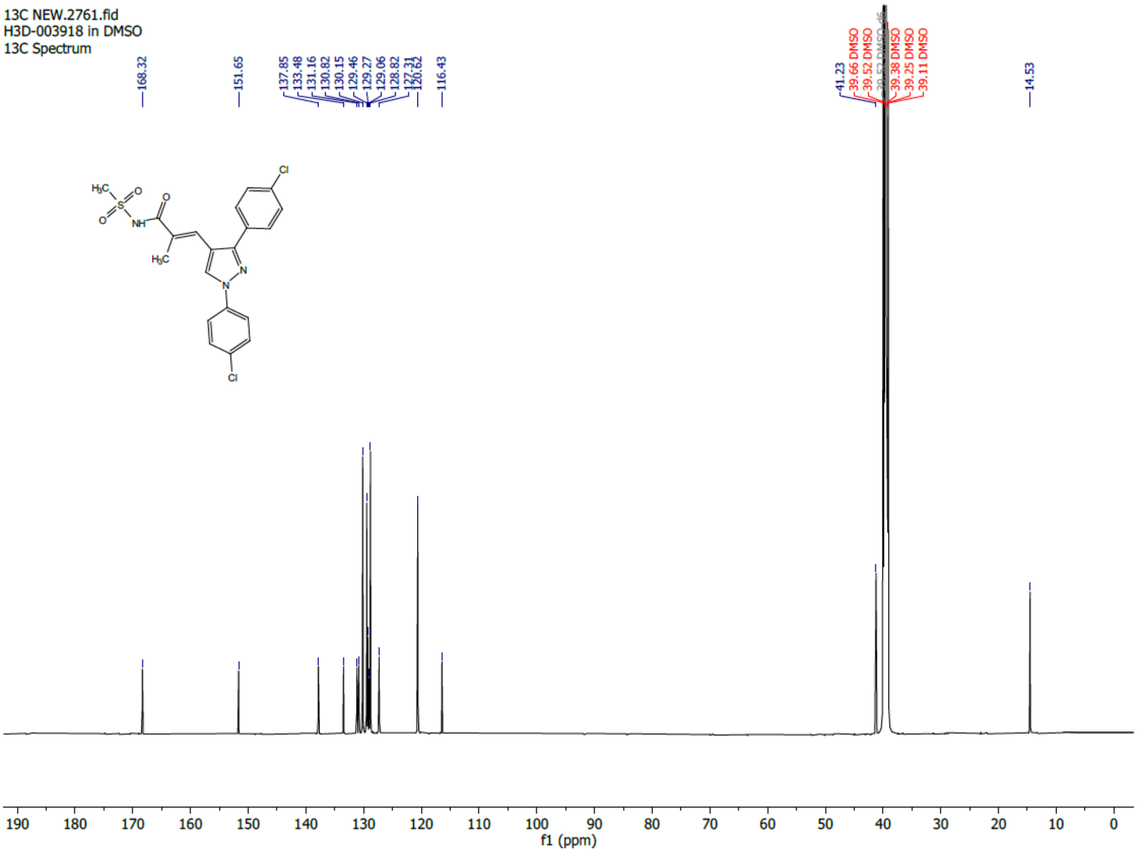

LC chromatogram of compound **6**

```
Additional Info : Peak(s) manually integrated
```

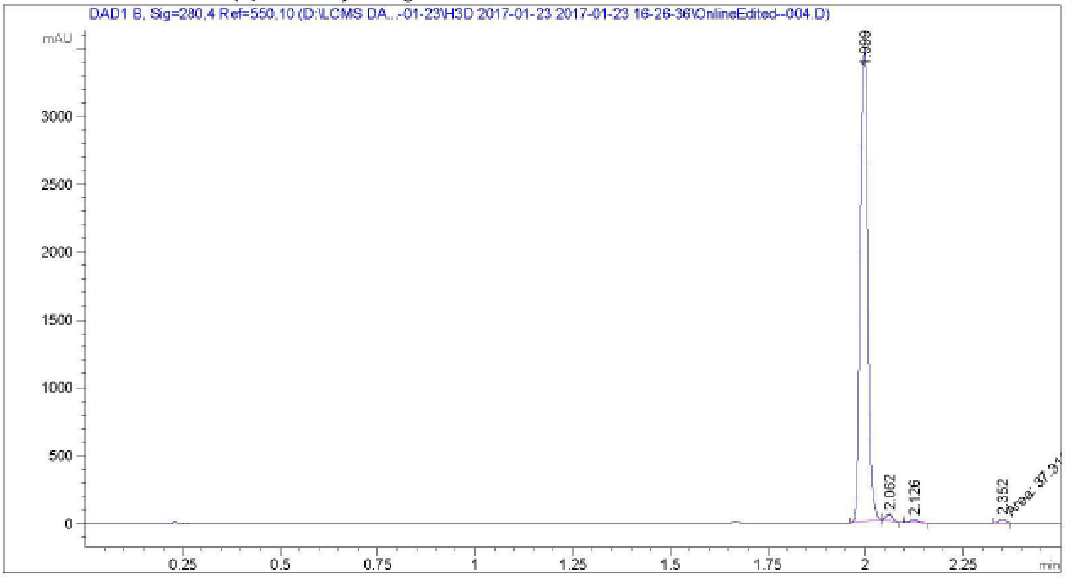


```
=====================================================================
                         Area Percent Report
=====================================================================

Sorted By             :      Signal
Multiplier            :      1.0000
Dilution              :      1.0000
Use Multiplier & Dilution Factor with ISTDs


Signal 1: DAD1 B, Sig=280,4 Ref=550,10

Peak RetTime Type  Width     Area      Height     Area
  #   [min]        [min]   [mAU*s]     [mAU]        %
----|-------|----|-------|----------|----------|--------|
   1   1.999 BB    0.0216 4623.76758 3453.33862 97.4291
   2   2.062 BB    0.0196   54.13139   46.36272  1.1406
   3   2.126 BB    0.0216   30.55790   22.87267  0.6439
   4   2.352 MM    0.0202   37.31810   30.80465  0.7863
```

Synthesis of 3-(1-(4-bromophenyl)-3-(4-chlorophenyl)-1*H*-pyrazol-4-yl)-*N*-(*tert*-butylsulfonyl)propanamide (7)

Compound **7** was synthesized according to the same synthetic procedure described for compound **6** and **17**.[2] Compound **7** was isolated as a white solid. Yield: 22%. ^{1}H NMR (400 MHz, MeOD) δ 8.13 (d, *J* = 0.8 Hz, 1H), 7.74 – 7.67 (m, 4H), 7.65 – 7.58 (m, 2H), 7.49 – 7.42 (m, 2H), 3.02 (t, *J* = 7.2 Hz, 2H), 2.65 (t, *J* = 7.2 Hz, 2H), 1.31 (s, 9H). ^{13}C NMR (151 MHz, MeOD) δ 173.4, 152.0, 140.4, 135.2, 133.6 (2C), 133.3, 130.5 (2C), 129.8 (2C), 129.1, 121.5 (2C), 121.4, 120.5, 62.5, 37.9, 24.5 (3C), 20.7. HRMS: calculated for $C_{22}H_{24}BrClN_3O_3S$ [M+H]$^+$, 524.0405 found 524.0413; HPLC purity 97%.

^{1}H NMR spectrum of compound **7**

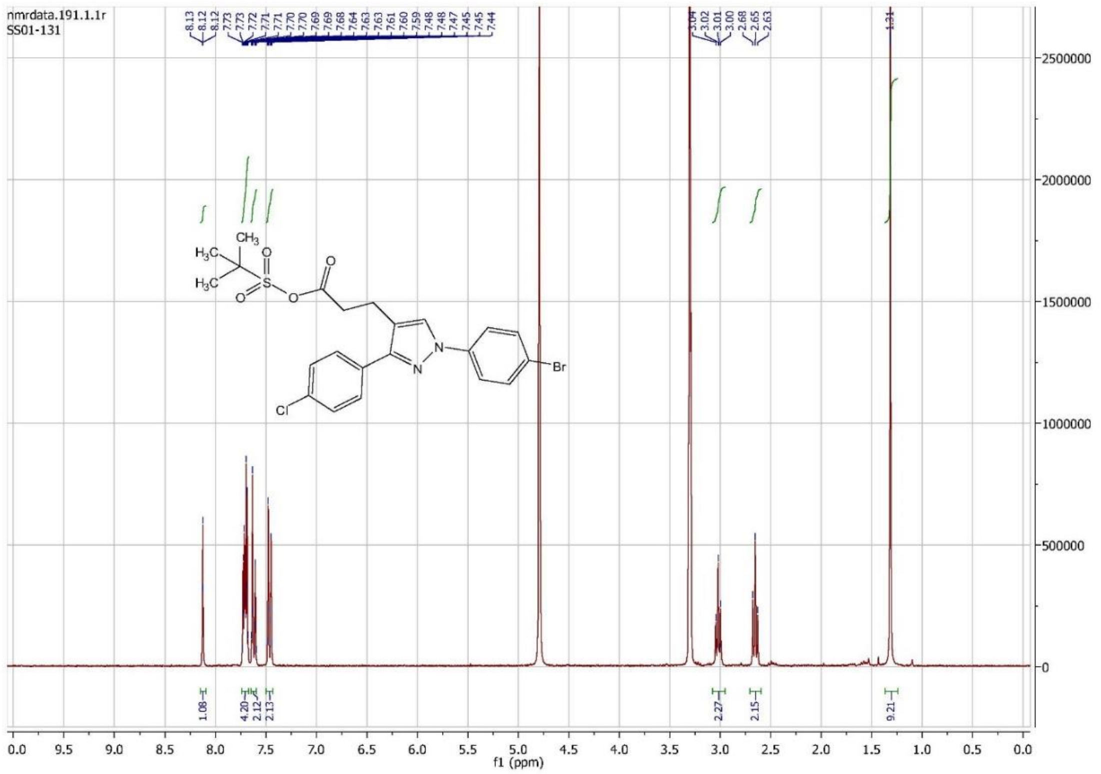

^{13}C NMR spectrum of compound **7**

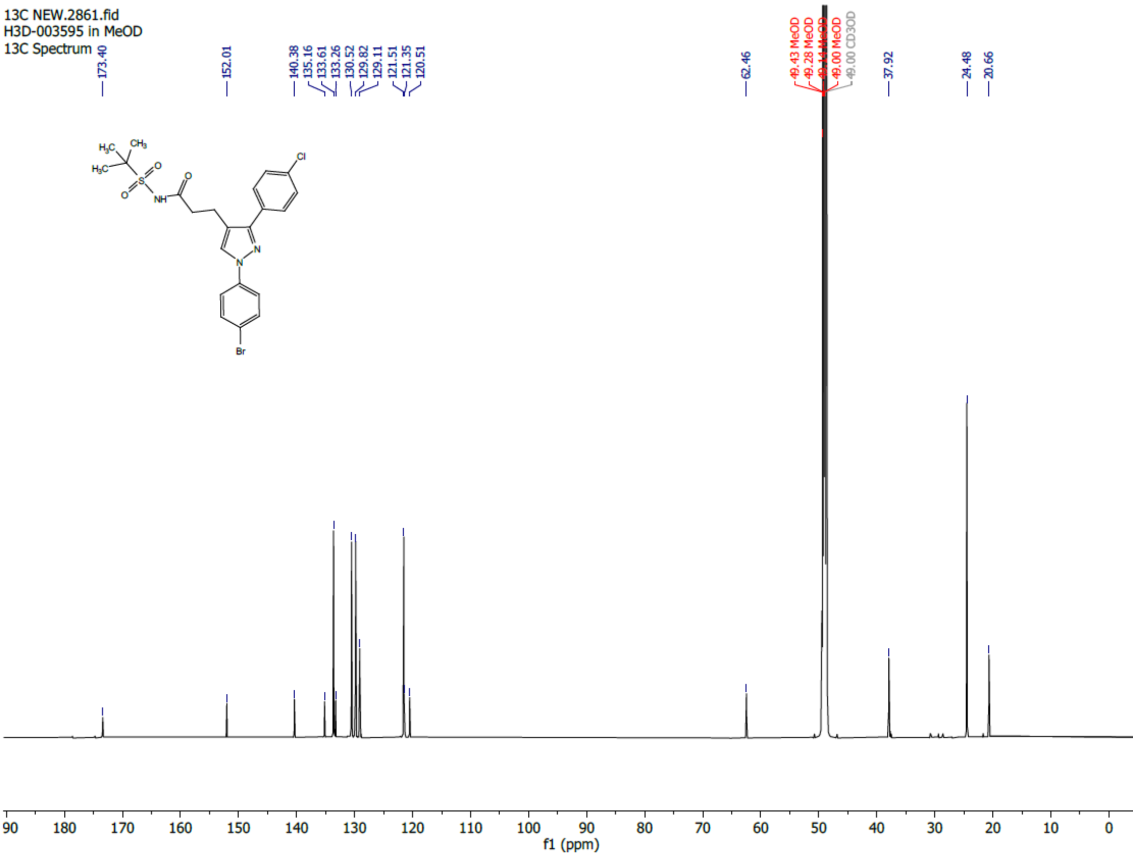

LC chromatogram of compound **7**

Additional Info : Peak(s) manually integrated

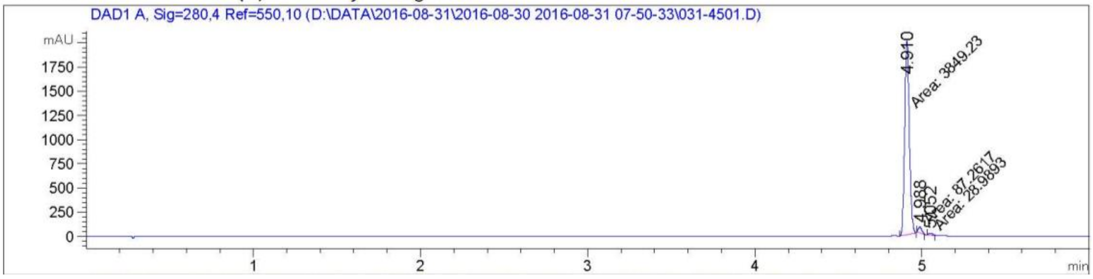

Signal 1: DAD1 A, Sig=280,4 Ref=550,10

Peak #	RetTime [min]	Type	Width [min]	Area [mAU*s]	Height [mAU]	Area %
1	4.910	MM	0.0320	3849.22949	2001.68262	97.0684
2	4.988	MM	0.0248	87.26167	58.57730	2.2005
3	5.052	MM	0.0257	28.98927	18.78742	0.7310

Totals : 3965.48043 2079.04733

Scheme S2: Synthetic pathway for compound 13.

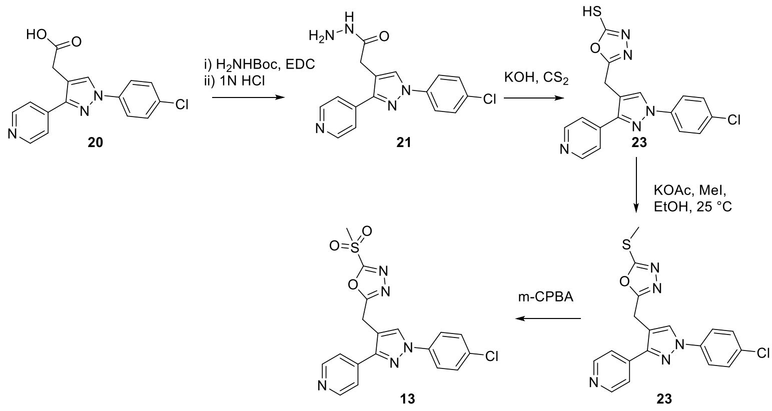

Synthesis of 2-(1-(4-chlorophenyl)-3-(pyridin-4-yl)-1*H*-pyrazol-4-yl)acetohydrazide (21): To a stirred solution of 2-(1-(4-chlorophenyl)-3-(pyridin-4-yl)-1*H*-pyrazol-4-yl)acetic acid **20** (3.0 g, 9.56 mmol), *tert*-butyl carbazate (2.5 g, 19.12 mmol) and diisopropylethyl amine (8.5 mL, 47.81 mmol) in dimethylformamide (25 mL), was added 1-ethyl-3-(3-dimethylaminopropyl)carbodiimide HCl (2.7 g, 14.34 mmol). The reaction mixture was stirred at room temperature for 16 h. Water was added and extracted with ethyl acetate (2 x 50 mL). The combined organic layer was washed with brine (1 x 50 mL) dried over sodium sulphate and concentrated under vacuum. The crude product was purified by column chromatography using 230-400 mesh silica gel with 30-50% ethyl acetate in petroleum ether to obtain Boc intermediate (2.8 g, 70%). The intermediate was dissolved in 1,4-dioxane (30 mL) and 4N HCl in 1,4-dioxane (10 mL) was added and stirred for 3 h. The solvent was evaporated under vacuum to **21** (1.4 g, 67%). ^{1}H NMR (400 MHz, DMSO-d_6): δ 11.56 (s, 1H), 8.93 (d, *J* = 5.60 Hz, 2H), 8.74 (s, 1H), 8.33 (d, *J* = 5.20 Hz, 2H), 7.99 (d, *J* = 5.20 Hz, 2H), 7.64 (d, *J* = 9.20 Hz, 2H), 3.93 (s, 2H), 3.16 (d, *J* = 5.20 Hz, 2H), LC-MS APCI: MS-ES$^+$ [M+H]$^+$ = 328.1.

Synthesis of 5-((1-(4-chlorophenyl)-3-(pyridin-4-yl)-1*H*-pyrazol-4-yl)methyl)-1,3,4-oxadiazole-2-thiol (22): 2-(1-(4-chlorophenyl)-3-(pyridin-4-yl)-1*H*-pyrazol-4-yl)acetohydrazide **21** (1.4 g, 4.27 mmol), carbon disulphide (0.52 mL, 8.54 mmol), potassium hydroxide (0.7 g, 12.81 mmol) and ethanol (20 mL) were taken in a sealed tube and heated to 90 °C for 16 h. Ethanol was evaporated under vacuum. The residue was diluted with water and pH adjusted to 1-2 with dilute HCl. The solids formed were filtered and dried under vacuum to obtain **22** (0.6 g, Crude). MS-ES$^+$ [M+H]$^+$ = 370.0. The crude product was taken as such for next step.

Synthesis of 2-((1-(4-chlorophenyl)-3-(pyridin-4-yl)-1*H*-pyrazol-4-yl)methyl)-5-(methylthio)-1,3,4-oxadiazole (23) : To a solution of 5-((1-(4-chlorophenyl)-3-(pyridin-4-yl)-1H-pyrazol-4-yl)methyl)-1,3,4-oxadiazole-2-thiol **22** (0.5 g, 1.35 mmol) and potassium acetate (0.26 g, 2.70 mmol) in ethanol (10 mL), was added methyl iodide (0.12 mL, 2.02 mmol) at 0°C. The reaction mixture was stirred at room temperature for 16 h. The solvent was evaporated under vacuum and the residue was taken for column purification using 230-400 mesh silica gel with 1-2% methanol in dichloromethane as eluent to obtain 2-((1-(4-chlorophenyl)-3-(pyridin-4-yl)-1*H*-pyrazol-4-yl)methyl)-5-(methylthio)-1,3,4-oxadiazole **23** (0.18 g, 35%). ^{1}H NMR (400 MHz, $CDCl_3$): δ 8.74 (d,

J = 4.64 Hz, 2H), 8.08 (s, 1H), 7.05-7.73 (m, 4H), 7.48 (d, *J* = 6.84 Hz, 2H), 4.30 (s, 2H), 2.78 (s, 3H). MS-ES$^+$ [M+H]$^+$ = 384.0.

Synthesis of 2-((1-(4-chlorophenyl)-3-(pyridin-4-yl)-1*H*-pyrazol-4-yl)methyl)-5-(methylsulfonyl)-1,3,4-oxadiazole (13): To a stirred solution of 2-((1-(4-chlorophenyl)-3-(pyridin-4-yl)-1*H*-pyrazol-4-yl)methyl)-5-(methylthio)-1,3,4-oxadiazole **23** (0.18 g, 0.46 mmol) in dichloromethane (15 mL) was added *m*-chloroperbenzoic acid (0.18 g, 1.03 mmol) and stirred for 12 h at room temperature. The reaction mixture was washed with aqueous sodium bicarbonate solution (1 x 10 mL), water (1 x 10 mL), dried over sodium sulphate and concentrated under vacuum. The crude product was purified with preparative HPLC to yield 2-((1-(4-chlorophenyl)-3-(pyridin-4-yl)-1*H*-pyrazol-4-yl)methyl)-5-(methylsulfonyl)-1,3,4-oxadiazole **13** (15 mg, 8%). ^{1}H NMR (400 MHz, MeOD): δ 8.50 (s, 1H), 8.42 (d, *J* = 6.64 Hz, 2H), 8.03 (d, *J* = 6.56 Hz, 2H), 7.89 (d, *J* = 8.56 Hz, 2H), 7.55 (d, *J* = 8.56 Hz, 2H), 4.62 (s, 2H), 3.21 (s, 3H). ^{13}C NMR (151 MHz, MeOD) δ 157.0, 148.2, 145.0, 140.5, 139.6, 135.7, 134.3, 133.8, 132.0, 131.3, 130.8, 130.7, 126.1, 121.8, 121.5, 115.4, 48.7 (overlapped with MeOD), 23.2. HRMS: calculated for $C_{18}H_{14}ClN_5O_3S$ [M+H]$^+$, 416.0579 found 416.0565; HPLC purity 93.8%.

^{1}H NMR spectrum of **13**

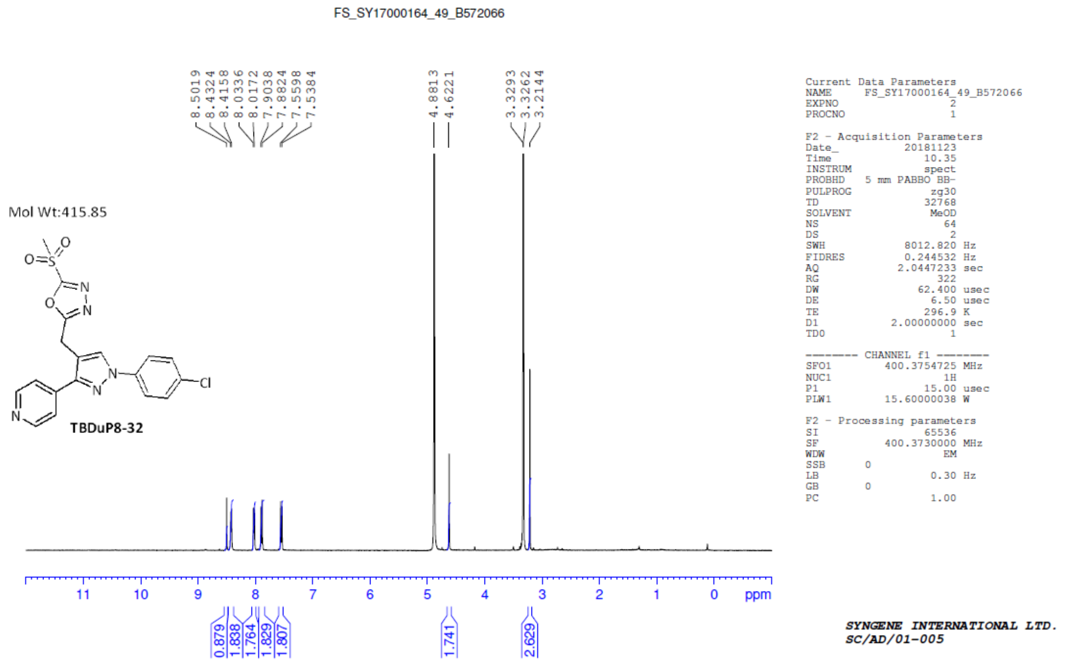

^{13}C NMR spectrum of **13**

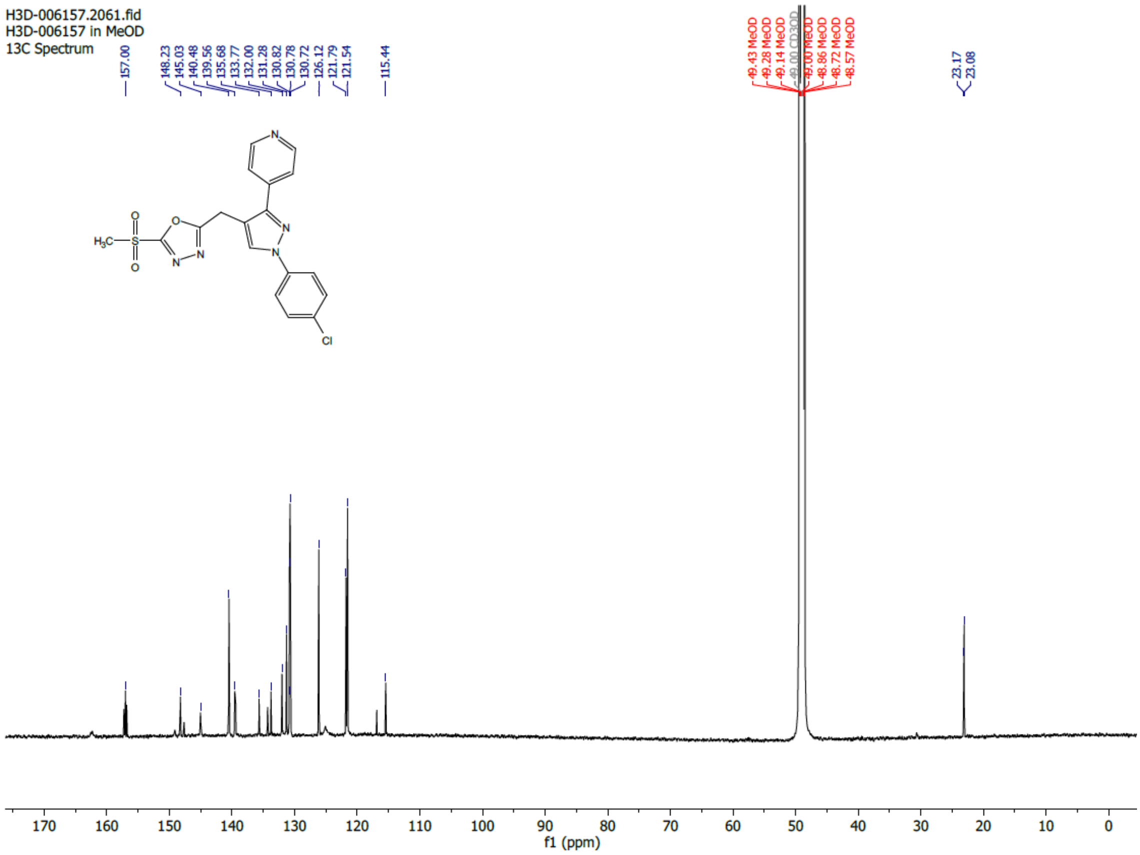

LC chromatogram of **13**

```
Data file       :  D:\DATA\NOV-2018\211118\B572066.D
Acq Method      :  D:\DATA\NOV-2018\211118\ZX_595FA.M
Injection Date  :  21 -Nov-2018                 Vial No.        : D1F-D3
Injection Time  :  12:35:00                     Injection vol   : 1.0 µL
Sample Name     :  FS_SY17000164_49_B572066

Method info  :     Column :ZORBAX XDB C-18 (50x4.6mm) 3.5 µm
                   Mobile Phase :A :0.1% HCOOH in H2O:ACN(95:5)
                   Mobile phase  :B:ACN
                   Flow Rate :1.5ml/min
                   Time (min)    %B
                   0.0            5
                   2.5           95
                   4.0           95
                   4.5            5
                   6.0            5
```

Mol Wt:415.85

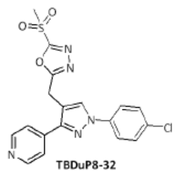

TBDuP8-32

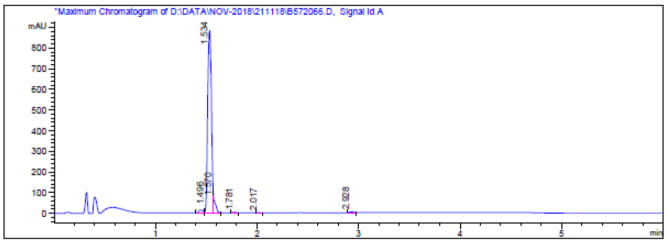

Peak No	RT min	Area	Area %
1	1.496	35.596	1.481
2	1.534	2253.681	93.781
3	1.570	95.491	3.974
4	1.781	5.717	0.238
5	2.017	2.318	0.096
6	2.928	10.318	0.429

Scheme S3. Synthetic pathway for compound 15.

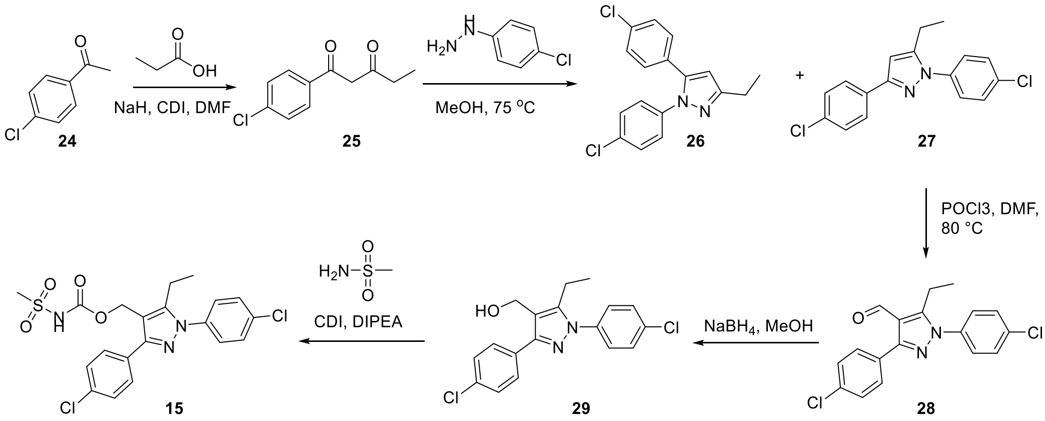

1-(4-chlorophenyl)pentane-1,3-dione (25): A solution of propionic acid (1.01 mL, 13.50 mmol) and CDI (3.38 g, 20.25 mmol) in anhydrous DMF (10 mL) was allowed to stir at room temperature under N_2 atmosphere for 3 hours. The mixture was added portion wise to a vigorous stirring suspension of 1-(4-chlorophenyl)ethan-1-one (**24**, 1.751 mL, 13.50 mmol) and sodium hydride 60% suspension in mineral oil (1.620 g, 40.5 mmol) in DMF (10 mL). The resulting reaction mixture was allowed to stir at 80 °C under N_2 atmosphere for 15 hours. The reaction mixture was allowed to cool at room temperature and poured into ice-cold and neutralised with 2 N HCl. The obtained aqueous layer was extracted with EtOAc (4 x 25 mL). The combined organic layers were washed with water, followed by brine, dried over $MgSO_4$, filtered and solvent evaporated in vacuo to yield a brown residue. The obtained residue was adsorbed into silica gel and purified using an ISCO Teledyne CombiFlash system (40 g SiO_2 cartrige) eluting a gradient of EtOAc in hexane (hexane 100% to hexane/EtOAc 95:5). Pure fractions were combined to yield **25** (0.492 g, 2.195 mmol, 16% yield) a yellow liquid. Intermediate used in the next step without further purification. MS-ES$^+$ $[M+H]^+$ = 211.0

1,3-*bis*(4-chlorophenyl)-5-ethyl-1*H*-pyrazole (27): To a stirring solution of **28** (0.693 g, 3.29 mmol) in methanol (40 mL) was added (4-chlorophenyl)hydrazine, Hydrochloride (0.589 g, 3.29 mmol). The resulting reaction mixture was allowed to reflux at 75 °C for 17 hours. LC-MS revealed clear product formation. The solvent was removed in vacuo to yield a residue which was adsorbed into silica gel and purified using an ISCO Teledyne CombiFlash system (25 g SiO_2 cartrige) eluting a gradient of EtOAc in hexane (hexane 100% to hexane/EtOAc 95:5) to yield two isomers, early fractions afforded the minor isomer **27** (0.236 g, 0.655 mmol, 20 % yield) as a yellow oil and later fractions afforded 1,5-bis(4-chlorophenyl)-3-ethyl-1*H*-pyrazole (**26**, 0.378 g, 1.180 mmol, 36 % yield) as a yellow oil. Intermediates used in the next step without further purification.

Data for 27: ^{1}H NMR (300 MHz, $CDCl_3$) δ 7.89 – 7.83 (m, 2H), 7.49 (d, *J* = 2.8 Hz, 4H), 7.41 (d, *J* = 8.5 Hz, 2H), 6.58 (s, 1H), 2.72 (q, *J* = 7.5 Hz, 2H), 1.31 (t, *J* = 7.5 Hz, 3H). MS-ES$^+$ $[M+H]^+$ = 317.0

Data for 26: ^{1}H NMR (300 MHz, $CDCl_3$) δ 7.38 – 7.29 (m, 4H), 7.25 – 7.12 (m, 4H), 6.40 (s, 1H), 2.85 (q, *J* = 7.6 Hz, 2H), 1.37 (t, *J* = 7.6 Hz, 3H). MS-ES$^+$ $[M+H]^+$ = 317.0.

1,3-*bis*(4-chlorophenyl)-5-ethyl-1*H*-pyrazole-4-carbaldehyde (28): To a stirring solution of **27** (236 mg, 0.744 mmol) in DMF (15 mL) was added $POCl_3$ (2.08 mL, 22.32 mmol) slowly at ambient temperature and the resulting reaction mixture was heated at 80 °C for 19 hours. The reaction mixture was cooled and poured onto ice-water and basified to pH=8 with NaOH. The obtained aqueous layer was extracted with EtOAc (4 x 15 mL). The combined organic layer was washed with H_2O (10 mL) followed by brine (10 mL), then dried over $MgSO_4$, filtered and solvent evaporated *in vacuo* to yield a crude product. The crude product was adsorbed into silica gel and purified using an ISCO Teledyne CombiFlash system (25 g SiO_2 cartridge) eluting a gradient of EtOAc in hexane (hexane 100% to hexane/EtOAc 85:15) to afford **28** (109 mg, 0.316 mmol, 42 % yield) as a yellow solid. Intermediate was used in the next step without further purification. ^{1}H NMR (300 MHz, $CDCl_3$) δ 10.05 (s, 1H), 7.66 (d, *J* = 8.7 Hz, 2H), 7.53 (d, *J* = 8.7 Hz, 2H), 7.49 – 7.39 (m, 4H), 2.99 (q, *J* = 7.5 Hz, 2H), 1.25 (t, *J* = 7.5 Hz, 3H). MS-ES$^+$ $[M+H]^+$ = 345.0.

(1,3-*bis*(4-chlorophenyl)-5-ethyl-1*H*-pyrazol-4-yl)methanol (29): In an ice-cold water bath, a solution of **28** (109 mg, 0.316 mmol) in methanol (15 ml) was added sodium borohydride (23.89 mg, 0.631 mmol) portion wise and the resulting reaction mixture was allowed to stir at room temperature for 1 hour. LC-MS indicated complete consumption of the starting material. The reaction was quenched by careful addition of H_2O (10 mL) and solvent was removed *in vacuo* to yield a residue. The residue obtained was diluted with water and extracted with EtOAc (4 x 15 mL), the combined organic layers were washed with brine (15 mL), dried over $MgSO_4$, filtered and solvent evaporated *in vacuo* to afford **29** (76 mg, 0.193 mmol, 61 % yield) as a yellow solid. Intermediate was used in the next step without further purification. MS-ES$^+$ $[M+H]^+$ = 347.0.

(1,3-*bis*(4-chlorophenyl)-5-ethyl-1*H*-pyrazol-4-yl)methyl (methylsulfonyl)carbamate (15): A pressure tube containing a solution of *N*-ethyl-*N*-isopropylpropan-2-amine (0.310 mL, 1.774 mmol), methanesulfonamide (42.2 mg, 0.443 mmol) and di(1*H*-imidazol-1-yl)methanone (71.9 mg, 0.443 mmol) in DCM (5 mL) was allowed to stir at room temperature for 16 hours. To this solution was added **28** (77 mg, 0.222 mmol) in DCM (5 mL) and the resulting reaction mixture was allowed to stir at 70 °C for 24 hours. At the end, the reaction mixture was cooled to room temperature, diluted with water and extracted with DCM (4 X 20 mL). The combined organic layers were washed with brine (20 mL), dried over $MgSO_4$, filtered and solvent evaporated *in vacuo* to afford a residue. The obtained residue was adsorbed into silica gel and purified using an ISCO Teledyne CombiFlash system (12 g SiO_2 cartridge) eluting a gradient of EtOAc in hexane (hexane 100% to hexane/EtOAc 30:70) to afford **15** (30 mg, 0.062 mmol, 28 % yield) as a white solid. ^{1}H NMR (300 MHz, MeOD) δ 7.71 (d, *J* = 8.5 Hz, 2H), 7.60 (d, *J* = 8.8 Hz, 2H), 7.52 (d, *J* = 8.8 Hz, 2H), 7.47 (d, *J* = 8.5 Hz, 2H), 5.23 (s, 2H), 3.21 (s, 3H), 2.88 (q, *J* = 7.6 Hz, 2H), 1.09 (t, *J* = 7.6 Hz, 3H). ^{13}C NMR (151 MHz, MeOD) δ 186.2, 160.0, 149.8, 147.9, 140.7, 139.2, 138.8 (2C), 137.9 (2C), 137.0 (2C), 136.9, 131.3, 128.8 (2C), 48.0, 41.0, 38.2, 30.6. HRMS: calculated for $C_{20}H_{20}Cl_2N_3O_4S$ $[M+H]^+$, 468.0546 found 468.0566. ; HPLC purity 97%.

^{1}H NMR spectrum of **15**

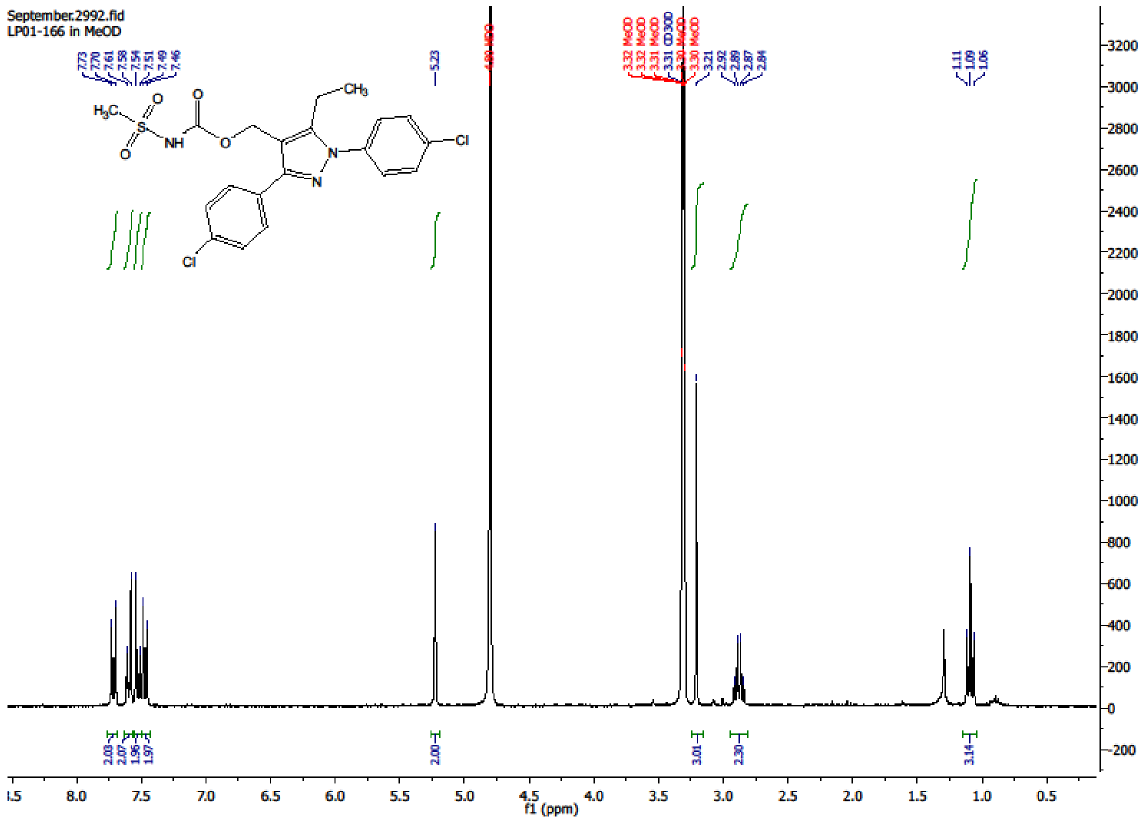

^{13}C NMR spectrum of **15**

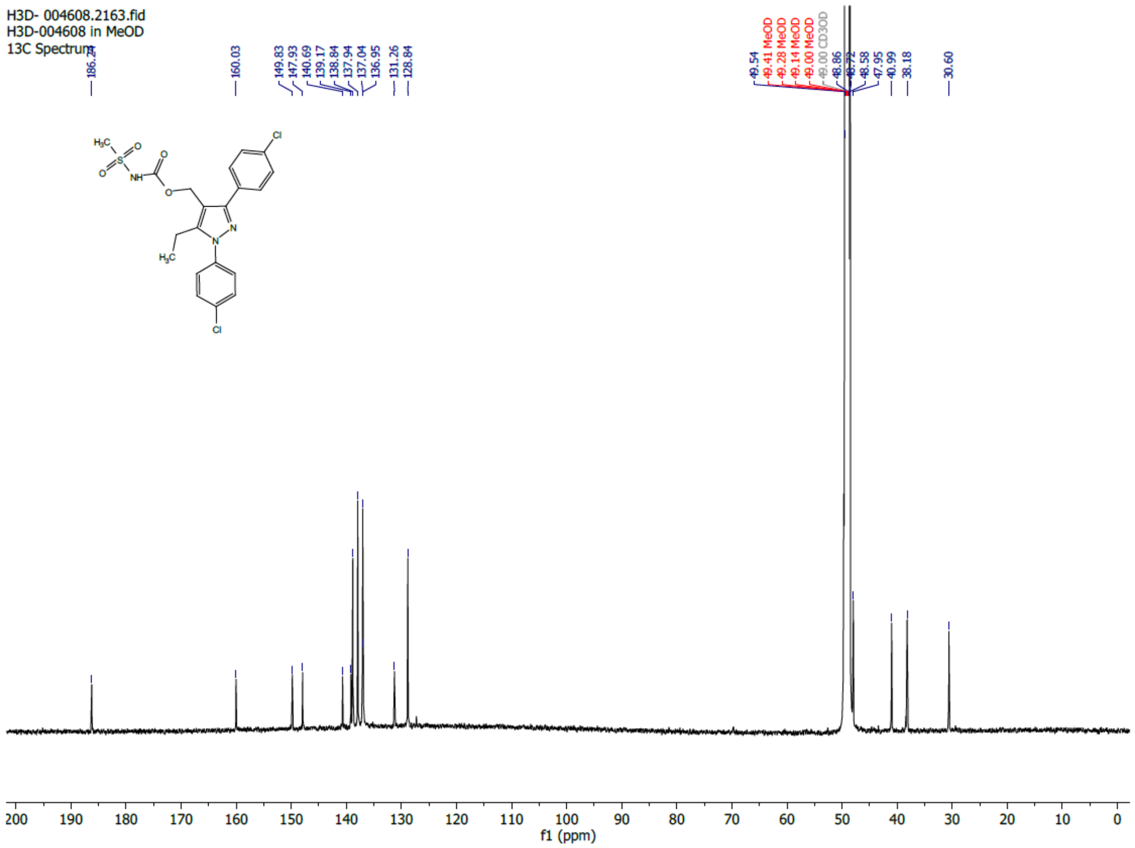

LC chromatogram of **15**

100-800m/z

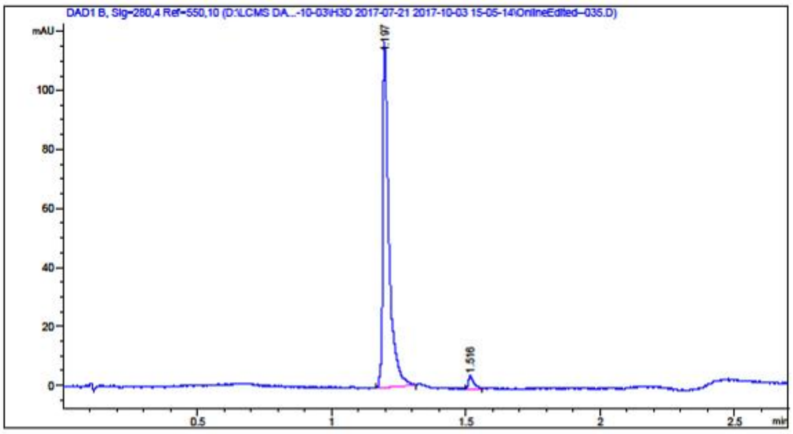

===

Area Percent Report

===

```
Sorted By            :      Signal
Multiplier           :      1.0000
Dilution             :      1.0000
Do not use Multiplier & Dilution Factor with ISTDs
```

Signal 1: DAD1 B, Sig=280,4 Ref=550,10

Peak #	RetTime [min]	Type	Width [min]	Area [mAU*s]	Height [mAU]	Area %
1	1.197	BB	0.0239	189.37801	116.77565	96.8256
2	1.516	BB	0.0209	6.20864	4.42996	3.1744

Totals : [illegible] [illegible]

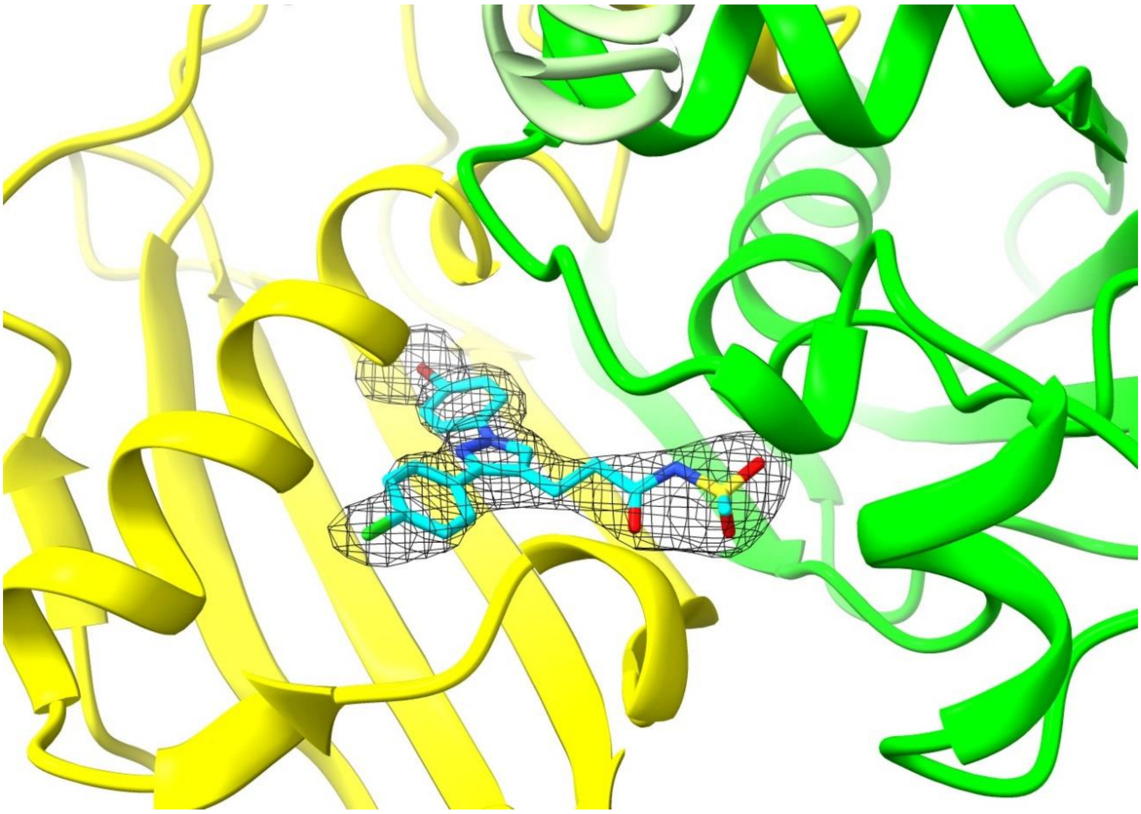

Figure S3. **Ligand Density map of the bound inhibitor 9 in Complex with *Mtb* HadAB -** Polder omit electron density F_o-F_c map for the ligand **9** bound to *Mtb* HadAB. Electron density is displayed as mesh, with a 5σ cutoff. The HadA subunit ribbon is colored yellow while the B subunit ribbon is colored green with the ligand carbons colored cyan.

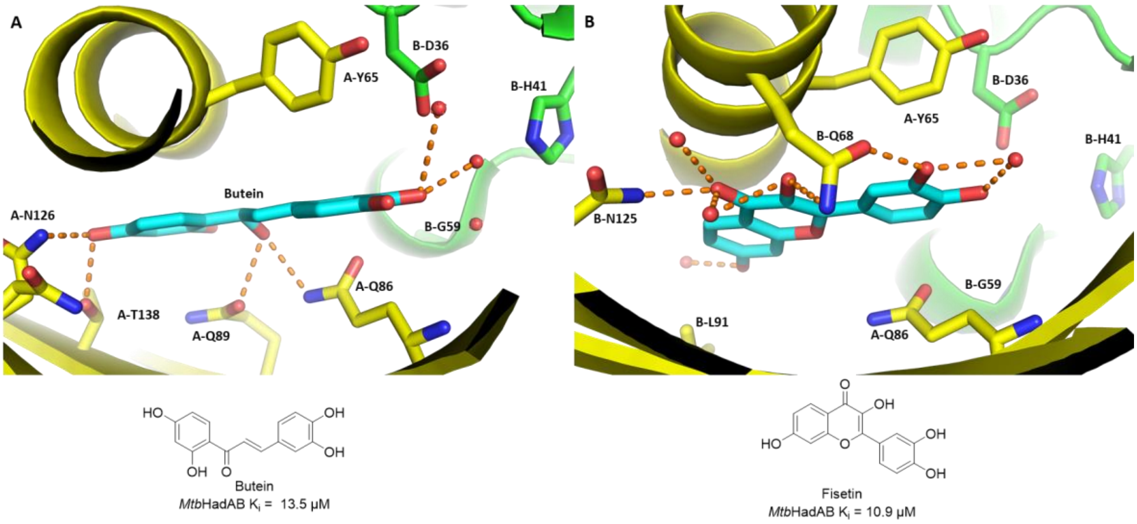

Figure S4. **3D illustrations of the binding modes of butein (A) and fisetin (B) the HadAB complex** - PDB ID: 4RLW and 4RLT respectively. Butein (A) interacts predominantly with the S1 subsite forming π-interactions with A-Y65 and hydrogen bonds to A-Q89 and A-Q86. Hydrogen bonds to A-N126 and A-T138 are present in the S4 subsite. Hydrogen bonds with two water molecules interacting with the catalytic dyad of B-D-36 and B-H41 are observed but no direct interactions with the catalytic hooks of the S2 main chain amides of A-Q86 and B-G59. These interactions are crucial for positioning the substrate for the dehydratase reaction catalysed by the B-D36/B-H41 dyad. (B) Fisetin, a coumarin also interacts predominantly with the S2 and S4 subsites. The coumarin group occupies the S4 cavity, forming hydrogen bonds with 3 water molecules, B-N125 and B-Q68. The dihydroxyphenyl group interacts with the S1 subsite via a π-interactions with A-Y65 and a hydrogen bond with lone water, not engaging with the water network around the catalytic dyad or the catalytic hooks of the A-Q86 and B-G59 main chain amides.

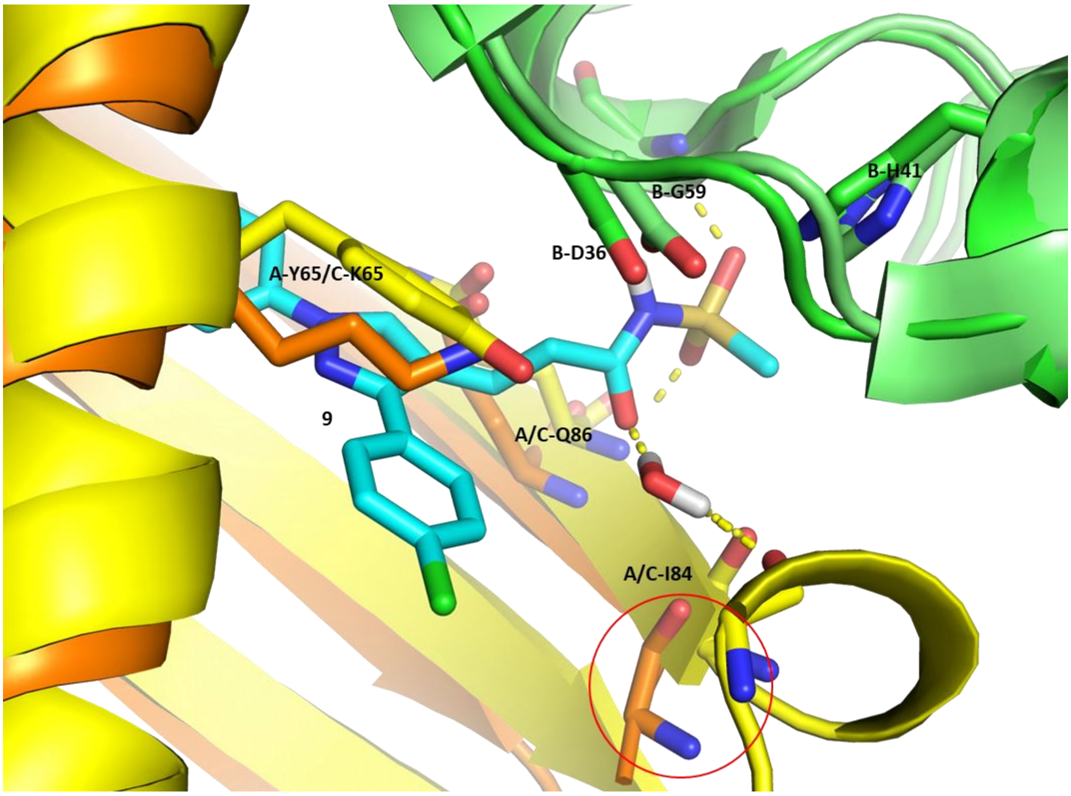

Figure S5. The unliganded structure or the *Mtb*HadBC (HadB -light green, HadC – orange) superimposed onto the crystal structure of *Mtb*HadAB (HadA – yellow, HadB lime green) in complex with **9** (turquoise). The two HadB subunits are shown to be identical while the HadC and HadB subunits are identical in the S2 subsite that interacts with the acyl sulfonamide moiety. The big difference occurs in the S3 subsite with the A-Y65 for C-K65 change. The apo form of the HadBC complex is also unsuitable for ligand docking due to the flexible C-K65 interfering with the ligand binding and a poorly defined region of the S2 subsite around C-I84 (circled in red).

References

(1) Pluskal, T.; Castillo, S.; Villar-Briones, A.; Orešič, M. MZmine 2: Modular framework for processing, visualizing, and analyzing mass spectrometry-based molecular profile data. *BMC Bioinformatics* **2010**, *11* (1), 395. DOI: 10.1186/1471-2105-11-395.

(2) Khonde, L. P.; Müller, R.; Boyle, G. A.; Reddy, V.; Nchinda, A. T.; Eyermann, C. J.; Fienberg, S.; Singh, V.; Myrick, A.; Abay, E.; Njoroge, M.; Lawrence, N.; Su, Q.; Myers, T. G.; Boshoff, H. I. M.; Barry, C. E.; Sirgel, F. A.; van Helden, P. D.; Massoudi, L. M.; Robertson, G. T.; Lenaerts, A. J.; Basarab, G. S.; Ghorpade, S. R.; Chibale, K. 1,3-Diarylpyrazolyl-acylsulfonamides as Potent Anti-tuberculosis Agents Targeting Cell Wall Biosynthesis in Mycobacterium tuberculosis. *Journal of Medicinal Chemistry* **2021**, *64* (17), 12790–12807. DOI: 10.1021/acs.jmedchem.1c00837.
